# Association of Cesarean Delivery With Risk of Neurodevelopmental and Psychiatric Disorders in the Offspring: A Systematic Review and Meta-analysis

**DOI:** 10.1001/jamanetworkopen.2019.10236

**Published:** 2019-08-28

**Authors:** Tianyang Zhang, Anna Sidorchuk, Laura Sevilla-Cermeño, Alba Vilaplana-Pérez, Zheng Chang, Henrik Larsson, David Mataix-Cols, Lorena Fernández de la Cruz

**Affiliations:** 1 Centre for Psychiatry Research, Department of Clinical Neuroscience, Karolinska Institutet, Stockholm, Sweden; 2 Stockholm Health Care Services, Stockholm County Council, Stockholm, Sweden; 3 Department of Medical Epidemiology and Biostatistics, Karolinska Institutet, Stockholm, Sweden; 4 Departamento de Medicina y Especialidades Médicas, Universidad de Alcalá, Madrid, Spain; 5 Departament de Personalitat, Avaluació i Tractaments Psicològics, Universitat de València, València, Spain; 6 School of Medical Sciences, Örebro University, Örebro, Sweden

## Abstract

**Importance:**

Birth by cesarean delivery is increasing globally, particularly cesarean deliveries without medical indication. Children born via cesarean delivery may have an increased risk of negative health outcomes, but the evidence for psychiatric disorders is incomplete.

**Objective:**

To evaluate the association between cesarean delivery and risk of neurodevelopmental and psychiatric disorders in the offspring.

**Data Sources:**

Ovid MEDLINE, Embase, Web of Science, and PsycINFO were searched from inception to December 19, 2018. Search terms included all main mental disorders in the *Diagnostic and Statistical Manual of Mental Disorders* (Fifth Edition).

**Study Selection:**

Two researchers independently selected observational studies that examined the association between cesarean delivery and neurodevelopmental and psychiatric disorders in the offspring.

**Data Extraction and Synthesis:**

Two researchers independently extracted data according to Preferred Reporting Items for Systematic Reviews and Meta-analyses (PRISMA) and Meta-analysis of Observational Studies in Epidemiology (MOOSE) reporting guidelines and assessed study quality using the Newcastle-Ottawa Scale. Random-effects meta-analyses were used to pool odds ratios (ORs) with 95% CIs for each outcome. Sensitivity and influence analyses tested the robustness of the results.

**Main Outcomes and Measures:**

The ORs for the offspring with any neurodevelopmental or psychiatric disorder who were born via cesarean delivery compared with those were born via vaginal delivery.

**Results:**

A total of 6953 articles were identified, of which 61 studies comprising 67 independent samples were included, totaling 20 607 935 deliveries. Compared with offspring born by vaginal delivery, offspring born via cesarean delivery had increased odds of autism spectrum disorders (OR, 1.33; 95% CI, 1.25-1.41; *I*^2^ = 69.5%) and attention-deficit/hyperactivity disorder (OR, 1.17; 95% CI, 1.07-1.26; *I*^2^ = 79.2%). Estimates were less precise for intellectual disabilities (OR, 1.83; 95% CI, 0.90-3.70; *I*^2^ = 88.2%), obsessive-compulsive disorder (OR, 1.49; 95% CI, 0.87-2.56; *I*^2^ = 67.3%), tic disorders (OR, 1.31; 95% CI, 0.98-1.76; *I*^2^ = 75.6%), and eating disorders (OR, 1.18; 95% CI, 0.96-1.47; *I*^2^ = 92.7%). No significant associations were found with depression/affective psychoses or nonaffective psychoses. Estimates were comparable for emergency and elective cesarean delivery. Study quality was high for 82% of the cohort studies and 50% of the case-control studies.

**Conclusions and Relevance:**

The findings suggest that cesarean delivery births are associated with an increased risk of autism spectrum disorder and attention-deficit/hyperactivity disorder, irrespective of cesarean delivery modality, compared with vaginal delivery. Future studies on the mechanisms behind these associations appear to be warranted.

## Key Points

**Question:**

Is birth by cesarean delivery associated with an increased risk of neurodevelopmental and psychiatric disorders in the offspring compared with birth by vaginal delivery?

**Findings:**

In this systematic review and meta-analysis of 61 studies comprising more than 20 million deliveries, birth by cesarean delivery was significantly associated with autism spectrum disorder and attention-deficit/hyperactivity disorder.

**Meaning:**

The findings suggest that understanding the potential mechanisms behind these associations is important, especially given the increase in cesarean delivery rates for nonmedical reasons.

## Teaser

This systematic review and meta-analysis examines the association between birth by cesarean delivery and risk of neurodevelopmental and psychiatric disorders in the offspring compared with birth by vaginal delivery.

## Introduction

Cesarean delivery can effectively reduce maternal and neonatal mortality and morbidity in the presence of complications, such as antepartum hemorrhage, fetal distress, abnormal fetal presentation, and hypertensive disease.^1^ During the past 3 decades, worldwide cesarean delivery rates have seen a more than 3-fold increase, from approximately 6% in 1990 to 21% in 2015, with substantial variations among and within countries.^1^ There is no precise information on appropriate cesarean delivery rates at the population level, although the international health care community considers 10% to 15% to be an optimal rate.^2^ Public health concerns have been raised because access to medically indicated cesarean delivery may be difficult in low-resource settings and unnecessary cesarean deliveries may be performed in high-resource settings.^1,3,4^

Despite being a life-saving procedure in the presence of complications, no evidence, to our knowledge, indicates that cesarean delivery, if not indicated, is beneficial for the offspring. On the contrary, previous studies have reported negative health outcomes in offspring born via cesarean delivery, including obesity,^5,6^ allergy,^6^ asthma,^6,7^ type 1 diabetes,^8^ and acute lymphoblastic leukemia.^9^ Cesarean delivery also has a potential association with early brain development. Previous studies have reported worse child cognitive development^10^ and higher rates of autism spectrum disorders (ASD) associated with cesarean delivery.^11^ Whether cesarean delivery is associated with increased risks of other neurodevelopmental and psychiatric disorders is unclear.

Previous studies^4,10,12^ have discussed the hypothesized biological mechanisms that may explain the associations between cesarean delivery and negative health outcomes in the offspring. For example, cesarean delivery might alter immune development through the perturbation of bacterial colonization, disturbing immune and sensory activation through lack of stress response or modifying epigenetic regulation in DNA methylation.^4,10,12^ Moreover, these adverse effects might vary depending on whether a cesarean delivery is performed before the onset of labor (ie, elective cesarean delivery) or after (ie, emergency cesarean delivery).^13^ Elective cesarean delivery can be scheduled by obstetricians in the presence of medical indications^14^ (eg, breech presentation, cephalopelvic disproportion) or requested by the mother because of culture preferences, previous negative birth experience, or fear of birth,^15,16^ which may indicate a genetic vulnerability to factors associated with psychological changes, such as stress, and associated in turn with neurodevelopmental or psychiatric illness in the offspring. However, emergency cesarean delivery occurs in more traumatic situations (eg, severe fetal distress, preeclampsia) that are associated with multiple negative birth outcomes.^13^ Elective and emergency cesarean deliveries are characterized by different factors and thus may be differentially associated with neurodevelopmental and psychiatric outcomes.

To address these gaps in the literature, we conducted a systematic review and meta-analysis of observational studies to quantify the extent of the association between cesarean delivery and a range of neurodevelopmental and psychiatric outcomes compared with vaginal delivery. Moreover, we investigated whether type of cesarean delivery (elective or emergency) or assisted vaginal delivery (ie, involving use of vacuum or forceps), compared with unassisted vaginal delivery, were differentially associated with neurodevelopmental and psychiatric outcomes in the offspring.

## Methods

### Search Strategy

We reported this systematic review and meta-analysis in accordance with the Preferred Reporting Items for Systematic Reviews and Meta-analyses (PRISMA) and Meta-analysis of Observational Studies in Epidemiology (MOOSE) reporting guidelines. The study was preregistered with International Prospective Register of Systematic Reviews (PROSPERO identifier CRD42018108298) before data extraction and analyses. We searched Ovid MEDLINE, Embase, Web of Science, and PsycINFO from inception to December 19, 2018, without language, date, or location restrictions. The search strategy was developed in association with information specialists at the Karolinska Institutet Library. Search terms included all main mental disorders in the *Diagnostic and Statistical Manual of Mental Disorders* (Fifth Edition) (*DSM-5*). The search strategy for each database is detailed in eTable 1 in the Supplement. We checked reference lists of relevant reviews for additional studies.

### Study Selection

We included observational studies that allowed estimation of the associations between obstetric mode of delivery (cesarean vs vaginal delivery) and neurodevelopmental and psychiatric disorders in the offspring. Studies were included regardless of the method of exposure ascertainment (ie, self-report or birth records). To maximize the quality of the included work, we only included studies if the outcome diagnoses were assessed through structured interviews or using standardized diagnostic criteria (eg, *International Classification of Diseases*, *DSM*, or equivalent). Self-reported or caregiver-reported outcomes were excluded. We further excluded review articles, book chapters, conference abstracts, and dissertations. Articles in languages other than English were translated by a native speaker or using Google Translate. Two researchers (T.Z. and L.S.-C. or A.V.-P.) independently screened and selected the articles first on the basis of titles and abstracts and then by examining the full texts; discrepancies were resolved through discussion with a senior researcher (A.S.).

### Data Extraction and Quality Assessment

Two researchers (T.Z. and L.S.-C. or A.V.-P.) independently extracted the data using predesigned Excel spreadsheets (Microsoft Corp). Disagreements were resolved through discussion. Extracted variables included country; data source; study design; age of the offspring; exposure; method of exposure ascertainment; diagnosis; diagnostic instruments; covariates controlled for by adjustment or matching; sample size; whether the study reported elective and emergency cesarean delivery or assisted and unassisted vaginal delivery separately and study participants in each group; and risk estimates (eg, odds ratios [ORs], hazard ratios). If the original study reported data on several outcomes, information on each outcome was retrieved separately. If studies reported no effect size for association of interest, we calculated ORs from raw data. When duplicate data were identified, we extracted data from the largest sample size for relevant outcomes (characteristics of the articles excluded because of duplicate data are presented in eTable 2 in the Supplement).

We assessed the methodologic quality of each study using the Newcastle-Ottawa Scale.^17^ Two researchers (T.Z. and L.S.-C. or A.V.-P.) independently assessed and scored each study according to the preestablished criteria. We judged the study quality to be high if the score was at least 7 points (of a possible 9) or otherwise to be low.

### Statistical Analysis

First, we examined the association between birth by cesarean delivery vs vaginal delivery and each outcome in the offspring separately using random effects models.^18^ We reported the results obtained after pooling each individual study’s most-adjusted estimate as ORs with 95% CIs. Second, we used the same approach to explore the associations of elective cesarean delivery, emergency cesarean delivery, and assisted vaginal delivery compared with unassisted vaginal delivery with the risk of neurodevelopmental and psychiatric disorders in the offspring. If the original studies reported the results from the analyses of both population data and sibling comparisons,^19,20,21,22,23,24^ we retrieved the former to reduce heterogeneity related to potential differences in study populations. For 8 studies^19,20,21,22,23,24,25,26^ that did not report ORs but reported hazard ratios, we calculated crude ORs from the data reported in the article using a 2 × 2 table.

Statistical heterogeneity among studies was assessed using Q and *I*^2^ statistics. For the Q statistic, a 2-sided *P* < .10 was considered as representative of statistically significant heterogeneity, and *I*^2^ values of 25%, 50%, and 75% were regarded as low, moderate, and high heterogeneity, respectively.^27^ If 10 or more original studies were included in the meta-analysis for an outcome, univariate meta-regressions on publication year, cesarean delivery use proportion, study design, country income status, and exposure ascertainment were applied to assess whether they were responsible for the heterogeneity. We assessed the presence of potential publication bias for each outcome using Egger tests and visualized by funnel plots.^28^ If the Egger tests revealed a potential publication bias, we used the contour-enhanced funnel plot and Duval and Tweedie nonparametric trim and fill method to further test the data.^29^

Furthermore, we undertook subgroup analyses for any differences in the association between birth by cesarean delivery vs vaginal delivery and each outcome for the following 6 moderators: country status (high- vs middle-income countries, according to the World Bank),^30^ proportion of cesarean deliveries in the study (lower vs higher than 15% as a proxy indicator for unnecessary cesarean delivery),^2^ exposure ascertainment (medical records vs self-report), offspring sex, whether risk measures were adjusted for parental psychiatry history, and study quality (high vs low). The results of the subgroup analyses were reported if at least 3 original studies were present in each stratum. We also performed influence analyses (so-called leave-1-out analysis) by iteratively removing 1 study at a time to confirm that the findings were not influenced by any single study. Finally, we performed sensitivity analyses using the least-adjusted or crude estimates from each study to assess whether the pooled effect size was sensitive to adjustment strategy.

All analyses were performed in Stata, version 15.1 (StataCorp). A 2-sided *P* < .05 was considered to be statistically significant.

## Results

**Figure 1.**
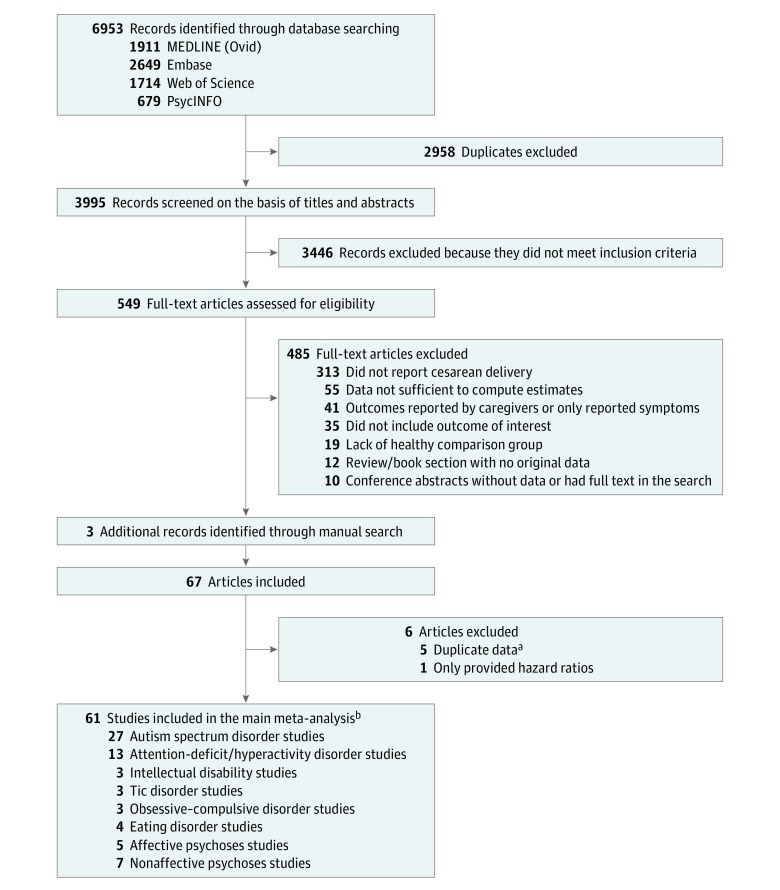
Preferred Reporting Items for Systematic Reviews and Meta-analyses (PRISMA) Flow Diagram ^a^Characteristics of these studies are presented in eTable 2 in the Supplement. ^b^Some studies address associations with more than 1 outcome.

We identified 6953 articles, of which 549 abstracts were selected for detailed assessment (Figure 1). A total of 61 studies comprising 67 independent samples and 20 607 935 deliveries met our inclusion criteria. The main characteristics of the included studies are presented in Table 1, and the variables adjusted and/or matched in each study are presented in eTable 3 in the Supplement. Of the 61 included studies, 27 studies^20,23,31,32,33,34,35,36,37,38,39,40,41,42,43,44,45,46,47,48,49,50,51,52,53,54,83^ reported on the association of cesarean delivery with a diagnosis of ASD (59 795 cases), 13 studies^19,24,42,52,55,56,57,58,59,60,61,62,63^ with attention-deficit/hyperactivity disorder (ADHD) (92 718 cases), 3 studies^42,64,65^ with intellectual disabilities (485 cases), 3 studies^22,66,67^ with tic disorders (6181 cases), 4 studies^25,26,68,69^ with eating disorders (4550 cases), 3 studies^21,70,71^ with obsessive-compulsive disorder (OCD; 7295 cases), 5 studies^72,73,74,75,76^ with major depression or affective psychoses (8561 cases), and 7 studies^73,77,78,79,80,81,82^ with nonaffective psychoses (7195 cases). Study quality was defined as high for 18 of 22 cohorts (82%) and 20 of 40 case-control studies (50%). Quality assessment summary scores are reported in Table 1.

**Table 1.** Characteristics of the Studies Included in the Main Meta-analysis

Source	Study			No. of Participants		Outcome(s)	Outcome Ascertainment	Age of Offspring	Newcastle -Ottawa Score
	Location	Period	Design	Cesarean Delivery	Vaginal Delivery				
**ASD**									
Matsuishi et al,^31^ 1999	Japan	1983-1987	Case-control	18	205	Autistic disorder	*DSM-III-R*	2-5 y	4
Mrozek-Budzyn et al,^32^ 2013	Poland	2007	Case-control	74	204	Childhood or atypical autism	*ICD-10* codes F84.0 and F84.1	2-15 y	5
Hamadé et al,^83^ 2013	Lebanon	2012	Case-control	31872	84 023	Autistic disorder	*DSM-IV-TR*	3-27 y	4
Maramara et al,^33^ 2014	United States	2000-2006	Case-control	31 872	84 023	Autistic disorder, PDD-NOS, or Asperger syndrome	*DSM-IV-TR*	NR	2
Burstyn et al,^34^ 2010	Canada	1998-2008	Cohort	49 456	170 572	ASD	*ICD-9* codes 299.0 and 299.8	4-10 y	8
Axelsson et al,^20^ 2019	Denmark	1997-2014	Cohort	119 433	560 440	ASD	*ICD-10* codes F84.0, F84.1, F84.5, F84.8	4-17 y	9
Maimburg and Vaeth,^35^ 2006	Denmark	1990-1999	Case-control	633	4099	Infantile autism	*ICD-8* code 299.0 and *ICD-10* code F84.0	Mean age at diagnosis, 4.6 y	8
Dodds et al,^36^ 2011	Canada	1990-2002	Cohort	26 754	102 948	ASD	*ICD-9* code 299 and *ICD-10* code F84	2-15 y	7
Chien et al,^37^ 2015	Taiwan	2004-2007	Cohort	174 376	362 297	Infantile autism	*ICD-9* code 299.0	2-6 y	9
Curran et al,^23^ 2015	Sweden	1982-2011	Cohort	340 108	2 357 206	ASD	*ICD-9* code 299 and *ICD-10* code F84	4-29 y	9
Guisso et al,^38^ 2018	Lebanon	NR	Case-control	125	189	ASD	*DSM-IV* and *DSM-5*	2-18 y	7
Yip et al,^39^ 2017	Norway	1984-2004	Cohort	Norway: 127 922; Finland: 156 863; WA: 65 953	Norway: 924 475; Finland: 891 684; WA: 279 228	ASD	*ICD-8*, *ICD-9*, *ICD-10,* and *DSM-IV*	5-25 y	9
	Finland	1987-2004							
	WA	1984-1999							
Schieve et al,^40^ 2014	United States	1994-2002	Case-control	NR	NR	ASD	*DSM-IV-TR*	8 y	6
		2000-2008							
Haglund and Källén,^41^ 2011	Sweden	1980-2005	Case-control	7434	61 530	Autistic disorder, childhood autism, Asperger syndrome	*DSM-III/IV* codes 299.00, 299.80; *ICD-10* codes F84.0, F98.5. Gillberg criteria	2.5-20 y	7
Chen et al,^42^ 2017	Taiwan	2005-2010	Cohort	6285	12 698	Autism	*DSM-5*	5.5 y	9
Polo-Kantola et al,^43^ 2014	Finland	1990-2007	Case-control	3349	17 117	Childhood autism, PDD, and Asperger syndrome	*ICD-9* code 299.x, *ICD-10* code F84.x	2-17 y	8
Duan et al,^44^ 2014	China	2011-2013	Case-control	287	285	Childhood autism	*DSM-IV* and Childhood Autism Rating Scale	3-6 y	5
Durkin et al,^45^ 2015	United States	1994-2008	Case-control	4624	26 843	ASD including autistic disorder, PDD-NOS, and Asperger syndrome	*DSM-IV-TR* and *ICD-9* codes	8 y	7
Eriksson et al,^46^ 2012	Sweden	2002-2008	Case-control	23 286	94 242	ASD	Clinical diagnosis	20-54 mo	5
Hultman et al,^47^ 2002	Sweden	1974-1993	Case-control	352	2096	Infantile autism	*ICD-9* code 299A	<10 y (mean age, 4.4 y for boys and 4.6 y for girls)	8
Kissin et al,^48^ 2015	United States	1996-2011	Cohort	27 152	15 231	Autistic disorder	*DSM-IV* code 299.0	5 y	6
Glasson et al,^49^ 2004	Australia	1980-1999	Case-control	380	1398	ASD including autism, PDD-NOS, and Asperger syndrome	*DSM-III* and *DSM-IV*	4-19 y	8
Zhang et al,^50^ 2010	China	2007	Case-control	77	104	Autism	*ICD-10* and Childhood Autism Rating Scale	3-21 y	5
El-Baz et al,^51^ 2011	Egypt	2008-2010	Case-control	89	213	Autism	*DSM-IV-TR*	2-13 y	4
Ji et al,^52^ 2018	United States	1998-2016	Case-control	214	434	ASD	*ICD-9* codes 299.0, 299.00, 299.01, 299.8, 299.80, 299.81, 299.9, 299.90, 299.91 and *ICD-10* codes F84.0, F84.8, F84.9	Median age at first ADHD diagnosis, 7 y	6
Winkler-Schwartz et al,^53^ 2014	Canada	1991-2013	Cohort	35	48	ASD	*DSM-IV*	3-17 y	5
Al-Jammas and Al-Dobooni,^54^ 2012	Iraq	2011-2012	Case-control	19	81	Autism, Asperger disorder, Rett syndrome	*DSM-IV-TR*	1.5-7 y	3
**ADHD**									
Çak and Gökler, ^55^ 2013	Turkey	2003-2008	Cohort	80	12	ADHD	K-SADS-PL and *DSM-IV*	5 y	5
Murray et al,^56^ 2016	United Kingdom, Brazil	ALSPAC:1991 to NR; Pelotas: 2004 to NR	Cohort	NR	NR	Any ADHD diagnosis including hyperactive-impulsive ADHD, inattentive ADHD, and combined ADHD	Development and well-being assessment based on *DSM-IV* criteria	7 y	7
Yeo et al,^57^ 2015	South Korea	2012-2013	Case-control	34	46	ADHD	*DSM-IV* and K-SADS-PL	6-12 y	5
Gustafsson and Källén,^58^ 2011	Sweden	1986-1996	Case-control	2996	29 016	ADHD	*DSM-III-R* and *DSM-IV*	Age at diagnosis, 5 to 17 y	6
Axelsson et al,^19^ 2019	Denmark	1997-2014	Cohort	117 863	553 727	ADHD or attention-deficit disorder	*ICD-8* code 308.01 or *ICD-10* codes F90 and F988 or ≥2 redeemed prescriptions for ADHD medication	4-17 y	8
Silva et al,^59^ 2014	Australia	1981-2003	Case-control	8863	34 829	ADHD	*DSM-IV* or *ICD-10*	4-25 y	8
Curran et al,^24^ 2016	Sweden	1990-2011	Cohort	238 687	1483 861	ADHD	*ICD-10* codes F90 and F98.8 or prescription of the psychostimulants methylphenidate (ATC code: N06BA04), amphetamine (N06BA01), dexamphetamine (N06BA02), or the noradrenergic reuptake inhibitor atomoxetine (N06BA09)	3-21 y	9
Sucksdorff et al,^60^ 2018	Finland	1991-2011	Case-control	8034	40 963	ADHD	*ICD-9* code 314 and *ICD-10* code F90	2-20 y	8
Chen et al,^42^ 2017	Taiwan	2005-2010	Cohort	6320	12 758	ADHD	*DSM-5*	5.5 y	9
Ketzer et al,^61^ 2012	Brazil	2001-2007	Case-control	NR	NR	ADHD–inattentive type	K-SADS-PL and *DSM-IV*	6-17 y	8
Halmøy et al,^62^ 2012	Norway	1967-2005	Case-control	69051	1 103 345	ADHD	*DSM-IV* or *ICD-10*	18-38 y	8
Ji et al,^52^ 2018	United States	1998-2016	Case-control	267	525	ADHD	*ICD-9* codes 314.00, 314.01, 314.1, 314.2, 314.8, or 314.9 or *ICD-10* codes F90.0, F90.1, F90.2, F90.8, or F90.9	Median age at first ADHD diagnosis, 7 y	6
Amiri et al,^63^ 2012	Iran	2009	Case-control	162	168	ADHD	K-SADS-PL	Mean age, 9.2 y for cases and 9.02 y for controls	5
**Intellectual Disabilities**									
Chen et al,^42^ 2017	Taiwan	2005-2010	Cohort	6360	12 825	Learning disabilities	*DSM-V*	5.5 y	9
Sussmann et al,^64^ 2009	United Kingdom	NR	Case-control	16	74	Intellectual disability	Wechsler Intelligence Scale and *ICD-10*	13-22 y	6
Bilder et al,^65^ 2013	United States	1994-2002	Case-control	2679	14 387	Learning disabilities	*ICD-9*	8 y	7
**Tic Disorders**									
Leivonen et al,^66^ 2016	Finland	1991-2010	Case-control	581	2961	Tourette syndrome	*ICD-9* code 3072D, *ICD-10* code F95.2	NR	8
Brander et al, ^22^ 2018	Sweden	1973-2013	Cohort	336 063	2 611 439	Tourette syndrome and chronic tic disorders	*ICD-8* code 306.2, *ICD-9* code 307C, and *ICD-10* codes F95.0, F95.1, F95.2, F95.8, F95.9	10-40 y	9
Cubo et al,^67^ 2014	Spain	2007-2009	Case-control	31	122	Tic disorder	*DSM-IV-TR*	6-16 y	7
**Eating Disorders**									
Razaz et and Cnattingius,^26^ 2018	Sweden	1992-2012	Cohort	53 807	428 768	Anorexia nervosa	*ICD-9* code 307B or *ICD-10* codes F500 and F501	10-20 y	8
Cnattingius et al,^68^ 1999	Sweden	1973-1984	Case-control	387	4299	Anorexia nervosa	*ICD-9* code 307B	10-21 y	7
Micali et al,^69^ 2015	United Kingdom	NR	Cohort	NR	NR	Eating disorders	Eating Disorders Examination Questionnaire	Mean age at assessment, 20.8 y	7
Hvelplund et al,^25^ 2016	Denmark	1997-2010	Cohort	173 937	727 290	Feeding and Eating disorder	*ICD-10* codes F98.2 and F50.8	0-48 mo	9
**OCD**									
Brander et al,^21^ 2016	Sweden	1973-2013	Cohort	248 840	2 137 846	OCD	*ICD-10* code F42	17-40 y	9
Geller et al,^70^ 2008	United States	NR	Case-control	47	132	OCD	K-SADS-PL, Children's Yale-Brown Obsessive Compulsive Scale, and *DSM-IV*	Mean age, 11.6 y	5
Vasconcelos et al,^71^ 2007	Brazil	NR	Case-control	47	91	OCD	SCID-I/P and K-SADS	11-44 y	3
**Affective Psychoses and Major Depressive Disorder**									
Hultman et al,^72^ 1999	Sweden	1973-1994	Case-control	89	1099	Affective psychoses	*ICD-9* code 296	15-21 y	7
O'Neill et al,^73^ 2016	Sweden	1982-2011	Cohort	125 356	1 215 881	Bipolar affective disorder, mania with psychotic symptoms, severe depressive episode with psychotic symptoms, and recurrent depressive disorder; current episode severe with psychotic symptoms	*ICD-10* codes F31, F30.2, F32.3, F33.3	16-29 y	9
Bain et al,^74^ 2000	United Kingdom	1971-2000	Case-control	NR	NR	Affective psychosis	*ICD-9* codes 296.0-296.9 and *ICD-10* codes F30, F31, F32.2, F32.3, F33.2, F33.3	18-26	5
Chudal et al,^75^ 2014	Finland	1983-2008	Case-control	320	1792	Bipolar disorder	*ICD-9* codes 2962, 2963, 2964, 2967 and *ICD-10* code F31.X	10-21 y	8
Gourion et al,^76^ 2008	Canada	1986-2005	Cohort	NR	NR	Major depressive disorder	*DSM-III-R* and *DSM-IV*	21 y	8
**Nonaffective Psychoses**									
Ordoñez et al,^77^ 2005	United States	NR	Cohort	NR	NR	Childhood-onset schizophrenia	K-SADS-PL and *DSM-IV*	12 y	3
Karlsson et al,^78^ 2012	Sweden	1975-2003	Case-control	85	679	Schizophrenia, schizoaffective disorders, persistent delusional disorders, induced delusional disorder, acute and transient psychotic disorders, unspecified nonorganic psychosis, schizotypal disorder	*DSM-IV* codes 295.x, 297.1, 297.3, 298.8, 298.9, 301.22; *ICD-9* codes 295.x, 297, 298 excluding A and B and *ICD-10* code F20-25, F28-29	<28 y	7
Jones et al,^79^ 1998	Finland	1966-1993	Case-control	47	1097	Schizophrenia	*DSM-III-R* codes 295.1, 295.2, 295.3, 295.6, or 295.9	16-28 y	8
Harrison et al,^80^ 2003	Sweden	1973-1997	Cohort	60 110	635 915	Nonaffective psychosis	*ICD-9* code 295, 297-298, *ICD-10* code F20-29	16-26 y	7
O'Neill et al,^73^ 2016	Sweden	1982-2011	Cohort	125 155	1 213 931	Schizophrenia, schizotypal disorder, persistent delusional disorders, acute and transient psychotic disorders, induced delusional disorder, schizoaffective disorders, other nonorganic psychotic disorders, and unspecified nonorganic psychosis	*ICD-10* codes F20-29	16-29 y	9
Kendell et al,^81^ 2000	United Kingdom	1971-1996	Case-control	NR	NR	Schizophrenia	*ICD-9* codes 295.0-259.9, *ICD-10* codes F20.0-20.3 and F20.5-20.9	18-25 y	7
Byrne et al,^82^ 2000	Ireland	1972-1992	Case-control	15	832	Schizophrenia	*ICD-9* codes 295.0-295.9	NR	5

Abbreviations: ADHD, attention-deficit/hyperactivity disorder; ALSPAC, Avon Longitudinal Study of Parents and Children; ASD, autism spectrum disorders; ATC, Anatomic Therapeutic Chemical; *DSM*, *Diagnostic and Statistical Manual of Mental Disorders*; *ICD*, *International Statistical Classification of Diseases and Related Health Problems*; K-SADS, Kiddie Schedule for Affective Disorders and Schizophrenia; K-SADS-PL, Schedule for Affective Disorders and Schizophrenia for School-Age Children—Present and Lifetime Versions; NR, not reported; OCD, obsessive-compulsive disorder; PDD-NOS, pervasive developmental disorder–not otherwise specified; SCID-I/P, Structured Clinical Interview for *DSM-IV*, Axis I disorders–patient edition; WA, Western Australia.

### Meta-analytic Association Between Cesarean Delivery and Neurodevelopmental and Psychiatric Disorders

**Figure 2.**
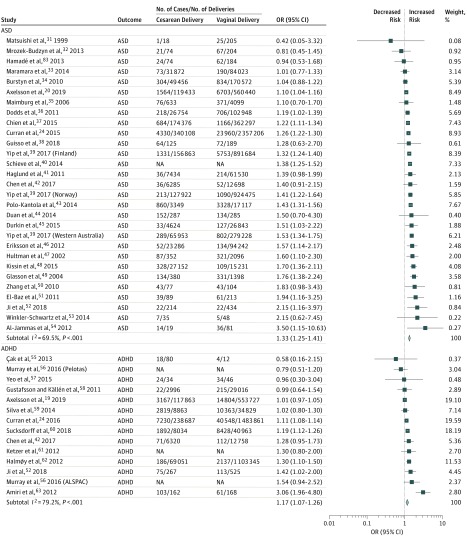
Forest Plot of the Results of Random-Effects Meta-analyses Stratified by Autism Spectrum Disorder (ASD) and Attention-Deficit/Hyperactivity Disorder (ADHD) Forest plot of odds ratios (ORs) in studies investigating the associations between cesarean delivery and each outcome. Diamonds show overall pooled estimate for each outcome. ALSPAC indicates Avon Longitudinal Study of Parents and Children; NA, not available.

**Figure 3.**
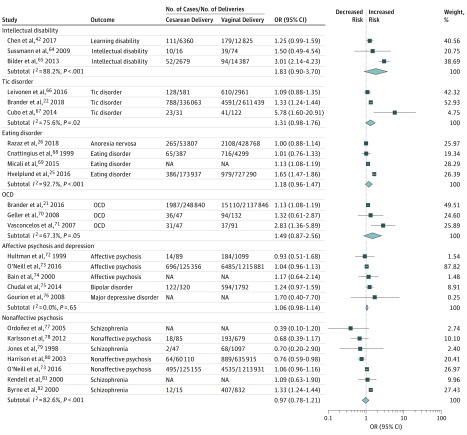
Forest Plot of the Results of Random-Effects Meta-analyses Stratified by Intellectual Disability, Tic Disorder, Eating Disorder, Obsessive-Compulsive Disorder (OCD), Affective Psychosis/Depression, and Nonaffective Psychosis Forest plot of odds ratios (ORs) in studies investigating the associations between cesarean delivery and each outcome. Diamonds show overall pooled estimate for each outcome. NA indicates not available.

Results of the meta-analysis for the first aim revealed that birth by cesarean delivery was significantly associated with increased odds of the offspring being diagnosed with ASD (OR, 1.33; 95% CI, 1.25-1.41) and ADHD (OR, 1.17; 95% CI, 1.07-1.26) compared with birth by vaginal delivery (Figure 2). The magnitude of the estimates was similar or higher for other neurodevelopmental and psychiatric outcomes, but the associations were not statistically significant (possibly because of the limited number of studies): intellectual disabilities (OR, 1.83; 95% CI, 0.90-3.70), OCD (OR, 1.49; 95% CI, 0.87-2.56), tic disorders (OR, 1.31; 95% CI, 0.98-1.76), and eating disorders (OR, 1.18; 95% CI, 0.96-1.47). The OR for depression/affective psychoses was 1.06 (95% CI, 0.98-1.14) and for nonaffective psychoses was 0.97 (95% CI, 0.78-1.21) (Figure 3).

For intellectual disabilities, OCD, tic disorders, and eating disorders, leave-1-out analysis revealed statistically significant associations with cesarean delivery births after omitting the following studies: Chen and colleagues^42^ (for intellectual disabilities; OR after exclusion, 2.61; 95% CI, 1.51-4.53), Vasconcelos and colleagues^71^ (for OCD; OR, 1.13; 95% CI, 1.08-1.19), and Cubo and colleagues^67^ (for tic disorders; OR, 1.24; 95% CI, 1.02-1.50) (eTable 4 in the Supplement). For the rest of the disorders, no individual studies influenced the results seen in the main analyses.

Study heterogeneity was high for ADHD (*I*^2^ = 79.2%), intellectual disabilities (*I*^2^ = 88.2%), tic disorders (*I*^2^ = 75.6%), and eating disorders (*I*^2^ = 92.7%); medium for ASD (*I*^2^ = 69.5%), OCD (*I*^2^ = 67.3%), and nonaffective psychosis (*I*^2^ = 82.6%); and low for depression and affective psychoses (*I*^2^ = 0.0%). Egger tests and funnel plots suggested a potential publication bias for ASD (eFigure 1 in the Supplement). In addition, a contour-enhanced funnel plot for ASD supported the Egger test, suggesting that the asymmetry was likely caused by publication bias (eFigure 2 in the Supplement). Furthermore, the Duval and Tweedie nonparametric trim and fill method for ASD led to a result similar to that obtained in the main analysis (OR, 1.32; 95% CI, 1.24-1.40).

### Meta-analytic Association Between Elective and Emergency Cesarean Delivery and Neurodevelopmental and Psychiatric Disorders

For the second aim, 20 studies^20,23,24,34,35,39,41,43,49,53,58,59,60,66,67,73,74,75,81,82^ (10 620 715 deliveries) and 18 studies^20,23,24,34,35,39,43,46,49,58,59,60,66,67,73,74,75,81^ (10 597 234 deliveries) assessed the risk of ASD, ADHD, tic disorders, and affective and nonaffective psychoses in offspring born via elective and emergency cesarean delivery, each compared with offspring born via unassisted vaginal delivery. Similar to the results of the main analyses, both elective and emergency cesarean deliveries were associated with increased odds of ADHD and ASD; for these analyses, heterogeneity was low (*I^2^* range, 10.7%-33.6%) except for ASD when assessing the association with elective cesarean delivery (*I*^2^ = 87.9%) (eFigure 3 in the Supplement). Elective and emergency cesarean deliveries were not associated with tic disorders or affective and nonaffective psychoses. In the 23 studies^22,23,24,26,36,41,43,45,47,49,51,58,59,60,66,67,68,73,75,79,81,82,84,85^ (8 415 429 deliveries) that assessed the associations with assisted vs unassisted vaginal delivery, we did not observe any association with any of the outcomes of interest except for tic disorders (OR, 1.28; 95% CI, 1.17-1.41; *I*^2^ = 0.0%) (eFigure 4 in the Supplement).

### Subgroup and Meta-regression Analyses

Results of our subgroup analyses for study design revealed that, compared with offspring born via vaginal delivery, the odds of offspring born via cesarean delivery being diagnosed with ASD or ADHD were significantly higher when pooling the results of case-control studies than when pooling those of cohort studies (Table 2). In studies that indicated unnecessary use of cesarean delivery (proportions ≥15%), we observed higher odds of ASD compared with studies with cesarean delivery proportions less than 15% (Table 2). Study quality scores and exposure assessment did not significantly affect the results (Table 2 and eTable 5 in the Supplement). Subgroup analyses on offspring sex and parental psychiatric history were not performed because of too few (<3) studies on each outcome with data available. Owing to data availability, we only performed meta-regression analyses for ASD and ADHD. Country income level and exposure ascertainment were associated with between-study variance for ADHD, but residual heterogeneity still remained significant (eTable 6 in the Supplement). None of the 5 factors examined (ie, publication year, proportion of cesarean deliveries, study design, country income status, and exposure ascertainment) were associated with heterogeneity or reduced residual heterogeneity. For our sensitivity analyses, we replicated all the analyses using crude or least-adjusted estimates from the same studies (eFigure 5 in the Supplement). No significant differences between the models were detected, although heterogeneity increased in the model including the least-adjusted estimates.

**Table 2.** Subgroup Analyses According to Proportion of Cesarean Deliveries, Study Quality Assessment, and Study Design for Neurodevelopmental and Psychiatric Disorders in Offspring Born via Cesarean Delivery Compared With Vaginal Delivery^a^

Outcome	Cesarean Delivery Use Proportion					Study Quality						Study Design				
	<15%		≥15%		*P* Value^b^	High (NOS Score ≥7)		Low (NOS Score <7)			*P* Value^b^	Cohort		Case-Control		*P* Value
	No. of Studies	OR (95% CI)	No. of Studies	OR (95% CI)		No. of Studies	OR (95% CI)	No. of Studies	OR (95% CI)			No. of Studies	OR (95% CI)	No. of Studies	OR (95% CI)	
ADHD	3	1.16 (1.03-1.31)	8	1.22 (1.05-1.42)^c^	.52	9	1.12 (1.05-1.21)^c^	5	1.37 (0.82-2.29)**^d^**	.68		6	1.07 (0.98-1.18)^c^	8	1.29 (1.10-1.52)^d^	.02
ASD	8	1.29 (1.24-1.34)	21	1.34 (1.22-1.48)^c^	.02	16	1.30 (1.22-1.39)^c^	13	1.43 (1.22-1.68)	.81		11	1.28 (1.19-1.37)^c^	18	1.41 (1.29-1.55)	<.001
Psychoses	9	1.00 (0.89-1.12)	3	1.03 (0.52-2.03)	>.99	9	1.02 (0.93-1.12)	3	1.14 (0.74-1.74)	.89		5	0.99 (0.88-1.12)	7	1.18 (1.00-1.39)	.81

Abbreviations: ADHD, attention-deficit/hyperactivity disorder; ASD, autism spectrum disorder; NOS, Newcastle-Ottawa Scale; OR, odds ratio.

^a^ Results of subgroup analyses on country income level and exposure ascertainment are reported in eTable 5 in the Supplement.

^b^ *P* values presented in the table are for group differences.

^c^ Study heterogeneity high (*I*^2^ > 75%; *P* < .05).

^d^ Study heterogeneity medium (*I*^2^ =  50%-75%; *P* < .05).

## Discussion

This systematic review and meta-analysis explored the association of cesarean delivery with a wide range of neurodevelopmental and psychiatric outcomes. Compared with vaginal delivery, cesarean delivery was associated with increased risk of several neurodevelopmental and psychiatric disorders. The estimates for ASD (33% increased odds) and ADHD (17% increased odds) were statistically significant, but other disorders, such as learning disabilities, tic disorders, OCD, and eating disorders, presented with similar or higher odds, although these were not statistically significant, possibly because of the modest number of studies. In the leave-1-out analysis, associations with intellectual disabilities, OCD, and tic disorders became statistically significant after the exclusion of single outlier studies. By contrast, the associations were close to the null and nonsignificant for depression and affective and nonaffective psychotic disorders, indicating some specificity. The association between cesarean delivery and offspring psychopathologic disorders contrasts with findings for assisted vaginal delivery (eg, forceps delivery), which was associated only with increased odds of tic disorders, whereas the association was close to the null with the remaining outcomes.

Our findings are consistent with previous meta-analyses conducted with a limited range of psychiatric outcomes, such as ASD^11^ or schizophrenia,^86^ and considerably expand the evidence by including all available data on a broad range of neurodevelopmental and psychiatric disorders. This inclusion was in part possible because of increased power afforded by the publication of large, nationwide registry-based studies on psychiatric disorders. For example, Curran and colleagues^11^ could not confirm an association between cesarean delivery and ADHD because of the availability of only 2 suitable studies at the time. We were able to include 13 studies on ADHD, of which 7 used data from population-based registries^19,24,58,59,60,62^ or national birth cohorts.^42^

For the first time, to our knowledge, we were able to examine elective cesarean delivery separately from emergency cesarean delivery in association with neurodevelopmental and psychiatric disorders. This distinction is theoretically important because it may hint at potentially different implicated mechanisms in the association between cesarean delivery and neurodevelopmental and psychiatric disorders. Of interest, the odds of ASD and ADHD in offspring born via elective and emergency cesarean delivery were nearly identical compared with unassisted vaginal delivery. The results for tic disorders and affective and nonaffective psychoses were less conclusive because of the few studies with small sample sizes. Of note, there have been no clear definitions of elective and emergency cesarean delivery in the literature, which may have influenced our results toward the null because of potential nondifferential exposure misclassification. Future studies should carefully define elective and emergency cesarean delivery, for example, using the Robson classification system.^87^

Subgroup analyses and meta-regressions (only performed for ADHD and ASD) suggested a potential association between higher rates of cesarean delivery and the presence of disorders in the offspring. Although it seems reasonable to assume that cesarean delivery is overused when exceeding a 15% rate of the total deliveries, additional studies are needed to investigate the reasons behind potentially unnecessary cesarean delivery to properly evaluate costs and benefits. Our subgroup analyses by type of study design found that the odds for ASD and ADHD were significantly weaker in cohort studies compared with case-control studies, indicating a potential bias in the main meta-analysis, in which studies of both designs were pooled together. This finding is also reflected in our quality assessment, in which cohort studies were more likely to be classified as high quality; however, subgroup analyses did not detect significant group differences between high- and low-quality studies. Future research should examine how potential bias, such as confounders, in particular indications for cesarean delivery, contribute to the observed associations.

### Strengths and Limitations

A strength of our review is the comprehensive scope of the literature search across multiple neurodevelopmental and psychiatric disorders, across 19 countries, in all languages, and with low publication bias. We used 4 different bibliographic databases and conducted the screening, data extraction, and quality assessment in duplicate. The main limitation of our study is the high level of heterogeneity in all outcomes except for depression and affective psychoses. Heterogeneity was decreased when we separated elective and emergency cesarean deliveries. Nevertheless, neither the subgroup nor meta-regression analyses could fully explain the high level of heterogeneity. Several other factors might have contributed to it. First, indication for cesarean delivery likely contributes to the heterogeneity among studies, but we were unable to explore the role of indications because of a lack of data in the original studies. The medical indications in the mothers may play an important role in the observed associations between cesarean delivery and the outcomes. Second, baseline age, age at diagnosis, and follow-up time substantially varied among studies and were not reported in some studies, which precluded us from exploring the role of these factors as contributors to heterogeneity. Third, methodologic differences in adjustment for parental, perinatal, and fetal factors in each study might account for the variance among studies. Studies with both population and sibling analyses suggest that the observed associations are likely attributable to familial confounding because the significant findings from the population-level analyses were attenuated in sibling comparisons.^20,21,23,36,59^ We therefore acknowledge the existence of unadjusted confounders and that the observed associations cannot be explained by only a single factor but are likely to be multifactorial.

## Conclusions

Our study findings suggest that birth by cesarean delivery is associated with certain neurodevelopmental and psychiatric disorders. The results appear to further add to the known adverse health outcomes associated with cesarean delivery and suggest judicious use of cesarean delivery.^1,3,4^ Statistical heterogeneity was high in the meta-analysis of some specific outcomes despite the use of strict inclusion criteria and our attempts to address the source of heterogeneity in subgroup analyses and meta-regression. This finding might suggest that other confounders, such as indication for cesarean delivery, could contribute to explaining some of the variation across studies. Future research should include further adjustment for potential confounders and consider genetically sensitive designs, such as sibling comparisons or twin and adoption studies. The mechanisms underlying the observed associations remain unknown and require empirical investigation to examine whether cesarean delivery plays a causal role in the development of neurodevelopmental and psychiatric disorders.

## Notes

Supplement. **eTable 1.** Full search strategy **eTable 2.** Characteristics of the articles excluded due to duplicate data **eTable 3.** Variables adjusted for or matched by in each included study in the main analysis **eTable 4.** Leave-one-study-out analyses **eTable 5.** Subgroup analyses according to country’s income level and exposure ascertainment for neurodevelopmental and psychiatric disorders in caesarean-born offspring compared to vaginal-born offspring **eTable 6.** Meta-regression analyses **eFigure 1.** Publication bias: Funnel plots and Egger’s tests for the main analyses **eFigure 2.** Contour-enhanced funnel plot for ASD for potential publication bias **eFigure 3.** Forest Plots of the Results of Random Effects Meta-analyses Stratified by Type of Cesarean Delivery (*Elective* or *Emergency*) and the Disorders **eFigure 4.** Results of the association between birth by assisted vaginal delivery (compared to *unassisted* vaginal delivery) and neurodevelopmental and psychiatric disorders in the offspring **eFigure 5.** Sensitivity analyses: crude or least adjusted models **eReferences**

## References

[1] Boerma T, Ronsmans C, Melesse DY, Global epidemiology of use of and disparities in caesarean sections. Lancet. 2018;392(10155):-. doi:10.1016/S0140-6736(18)31928-7 30322584

[2] Moore B Appropriate-technology for birth. Lancet. 1985;2(8458):787. doi:10.1016/S0140-6736(85)90673-7 2864526

[3] Betrán AP, Temmerman M, Kingdon C, Interventions to reduce unnecessary caesarean sections in healthy women and babies. Lancet. 2018;392(10155):1358-1368. doi:10.1016/S0140-6736(18)31927-5 30322586

[4] Sandall J, Tribe RM, Avery L, Short-term and long-term effects of caesarean section on the health of women and children. Lancet. 2018;392(10155):1349-1357. doi:10.1016/S0140-6736(18)31930-5 30322585

[5] Kuhle S, Tong OS, Woolcott CG Association between caesarean section and childhood obesity: a systematic review and meta-analysis. Obes Rev. 2015;16(4):295-303. doi:10.1111/obr.12267 25752886

[6] Keag OE, Norman JE, Stock SJ Long-term risks and benefits associated with cesarean delivery for mother, baby, and subsequent pregnancies: systematic review and meta-analysis. PLoS Med. 2018;15(1):e1002494. doi:10.1371/journal.pmed.1002494 29360829 PMC5779640

[7] Thavagnanam S, Fleming J, Bromley A, Shields MD, Cardwell CR A meta-analysis of the association between caesarean section and childhood asthma. Clin Exp Allergy. 2008;38(4):629-633. doi:10.1111/j.1365-2222.2007.02780.x 18352976

[8] Cardwell CR, Stene LC, Joner G, Caesarean section is associated with an increased risk of childhood-onset type 1 diabetes mellitus: a meta-analysis of observational studies. Diabetologia. 2008;51(5):726-735. doi:10.1007/s00125-008-0941-z 18292986

[9] Marcotte EL, Thomopoulos TP, Infante-Rivard C, Caesarean delivery and risk of childhood leukaemia: a pooled analysis from the Childhood Leukemia International Consortium (CLIC). Lancet Haematol. 2016;3(4):e176-e185. doi:10.1016/S2352-3026(16)00002-8 27063976 PMC5283076

[10] Polidano C, Zhu A, Bornstein JC The relation between cesarean birth and child cognitive development. Sci Rep. 2017;7(1):11483. doi:10.1038/s41598-017-10831-y 28904336 PMC5597642

[11] Curran EA, O’Neill SM, Cryan JF, Research review: birth by caesarean section and development of autism spectrum disorder and attention-deficit/hyperactivity disorder: a systematic review and meta-analysis. J Child Psychol Psychiatry. 2015;56(5):500-508. doi:10.1111/jcpp.12351 25348074

[12] Cho CE, Norman M Cesarean section and development of the immune system in the offspring. Am J Obstet Gynecol. 2013;208(4):249-254. doi:10.1016/j.ajog.2012.08.009 22939691

[13] Yang XJ, Sun SS Comparison of maternal and fetal complications in elective and emergency cesarean section: a systematic review and meta-analysis. Arch Gynecol Obstet. 2017;296(3):503-512. doi:10.1007/s00404-017-4445-2 28681107

[14] Mylonas I, Friese K Indications for and risks of elective cesarean section. Dtsch Arztebl Int. 2015;112(29-30):489-495. 26249251 10.3238/arztebl.2015.0489 PMC4555060

[15] Tschudin S, Alder J, Hendriksen S, Previous birth experience and birth anxiety: predictors of caesarean section on demand? J Psychosom Obstet Gynaecol. 2009;30(3):175-180. doi:10.1080/01674820902789233 19639511

[16] Finger C Caesarean section rates skyrocket in Brazil. Lancet. 2003;362(9384):628. doi:10.1016/S0140-6736(03)14204-3 12947949

[17] Wells GA, Shea B, O’Connell D, The Newcastle-Ottawa Scale (NOS) for assessing the quality of nonrandomised studies in meta-analyses. http://www.ohri.ca/programs/clinical_epidemiology/oxford.asp. Accessed August 25, 2018.

[18] Borenstein M, Hedges LV, Higgins JPT, Rothstein HR A basic introduction to fixed-effect and random-effects models for meta-analysis. Res Synth Methods. 2010;1(2):97-111. doi:10.1002/jrsm.12 26061376

[19] Axelsson PB, Clausen TD, Petersen AH, Investigating the effects of cesarean delivery and antibiotic use in early childhood on risk of later attention deficit hyperactivity disorder. J Child Psychol Psychiatry. 2019;60(2):151-159. doi:10.1111/jcpp.12961 30136734

[20] Axelsson PB, Clausen TD, Petersen AH, Relation between infant microbiota and autism? results from a national cohort sibling design study. Epidemiology. 2019;30(1):52-60. doi:10.1097/EDE.0000000000000928 30273187

[21] Brander G, Rydell M, Kuja-Halkola R, Association of perinatal risk factors with obsessive-compulsive disorder: a population-based birth cohort, sibling control study. JAMA Psychiatry. 2016;73(11):1135-1144. doi:10.1001/jamapsychiatry.2016.2095 27706475

[22] Brander G, Rydell M, Kuja-Halkola R, Perinatal risk factors in Tourette’s and chronic tic disorders: a total population sibling comparison study. Mol Psychiatry. 2018;23(5):1189-1197. doi:10.1038/mp.2017.31 28348386 PMC5984087

[23] Curran EA, Dalman C, Kearney PM, Association between obstetric mode of delivery and autism spectrum disorder: a population-based sibling design study. JAMA Psychiatry. 2015;72(9):935-942. doi:10.1001/jamapsychiatry.2015.0846 26107922

[24] Curran EA, Khashan AS, Dalman C, Obstetric mode of delivery and attention-deficit/hyperactivity disorder: a sibling-matched study. Int J Epidemiol. 2016;45(2):532-542. doi:10.1093/ije/dyw001 27063604

[25] Hvelplund C, Hansen BM, Koch SV, Andersson M, Skovgaard AM Perinatal risk factors for feeding and eating disorders in children aged 0 to 3 years. Pediatrics. 2016;137(2):e20152575. doi:10.1542/peds.2015-2575 26764360

[26] Razaz N, Cnattingius S Association between maternal body mass index in early pregnancy and anorexia nervosa in daughters. Int J Eat Disord. 2018;51(8):906-913. doi:10.1002/eat.22921 30051496

[27] Higgins JPT, Thompson SG, Deeks JJ, Altman DG Measuring inconsistency in meta-analyses. BMJ. 2003;327(7414):557-560. doi:10.1136/bmj.327.7414.557 12958120 PMC192859

[28] Egger M, Davey Smith G, Schneider M, Minder C Bias in meta-analysis detected by a simple, graphical test. BMJ. 1997;315(7109):629-634. doi:10.1136/bmj.315.7109.629 9310563 PMC2127453

[29] Duval S, Tweedie R Trim and fill: a simple funnel-plot-based method of testing and adjusting for publication bias in meta-analysis. Biometrics. 2000;56(2):455-463. doi:10.1111/j.0006-341X.2000.00455.x 10877304

[30] Fantom N, Serajuddin U The World Bank's classification of countries by income. Working paper WPS 7528. https://openknowledge.worldbank.org/handle/10986/23628. Accessed December 10, 2018.

[31] Matsuishi T, Yamashita Y, Ohtani Y, Brief report: incidence of and risk factors for autistic disorder in neonatal intensive care unit survivors. J Autism Dev Disord. 1999;29(2):161-166. doi:10.1023/A:1023048812202 10382137

[32] Mrozek-Budzyn D, Majewska R, Kieltyka A Prenatal, perinatal and neonatal risk factors for autism: study in Poland. Cent Eur J Med. 2013;8(4):424-430. doi:10.2478/s11536-013-0174-5

[33] Maramara LA, He W, Ming X Pre- and perinatal risk factors for autism spectrum disorder in a New Jersey cohort. J Child Neurol. 2014;29(12):1645-1651. doi:10.1177/0883073813512899 24413357

[34] Burstyn I, Sithole F, Zwaigenbaum L Autism spectrum disorders, maternal characteristics and obstetric complications among singletons born in Alberta, Canada. Chronic Dis Can. 2010;30(4):125-134. 20946713

[35] Maimburg RD, Vaeth M Perinatal risk factors and infantile autism. Acta Psychiatr Scand. 2006;114(4):257-264. doi:10.1111/j.1600-0447.2006.00805.x 16968363

[36] Dodds L, Fell DB, Shea S, Armson BA, Allen AC, Bryson S The role of prenatal, obstetric and neonatal factors in the development of autism. J Autism Dev Disord. 2011;41(7):891-902. doi:10.1007/s10803-010-1114-8 20922473

[37] Chien L-N, Lin H-C, Shao Y-HJ, Chiou S-T, Chiou H-Y Risk of autism associated with general anesthesia during cesarean delivery: a population-based birth-cohort analysis. J Autism Dev Disord. 2015;45(4):932-942. doi:10.1007/s10803-014-2247-y 25256350

[38] Guisso DR, Saadeh FS, Saab D, Association of autism with maternal infections, perinatal and other risk factors: a case-control study. J Autism Dev Disord. 2018;48(6):2010-2021. doi:10.1007/s10803-017-3449-x 29332178

[39] Yip BHK, Leonard H, Stock S, Caesarean section and risk of autism across gestational age: a multi-national cohort study of 5 million births. Int J Epidemiol. 2017;46(2):429-439. 28017932 10.1093/ije/dyw336 PMC5837358

[40] Schieve LA, Tian LH, Baio J, Population attributable fractions for three perinatal risk factors for autism spectrum disorders, 2002 and 2008 autism and developmental disabilities monitoring network. Ann Epidemiol. 2014;24(4):260-266. doi:10.1016/j.annepidem.2013.12.014 24529515 PMC4562459

[41] Haglund NG, Källén KB Risk factors for autism and Asperger syndrome: perinatal factors and migration. Autism. 2011;15(2):163-183. doi:10.1177/1362361309353614 20923887

[42] Chen G, Chiang WL, Shu BC, Guo YL, Chiou ST, Chiang TL Associations of caesarean delivery and the occurrence of neurodevelopmental disorders, asthma or obesity in childhood based on Taiwan birth cohort study. BMJ Open. 2017;7(9):e017086. doi:10.1136/bmjopen-2017-017086 28963295 PMC5623585

[43] Polo-Kantola P, Lampi KM, Hinkka-Yli-Salomäki S, Gissler M, Brown AS, Sourander A Obstetric risk factors and autism spectrum disorders in Finland. J Pediatr. 2014;164(2):358-365. doi:10.1016/j.jpeds.2013.09.044 24183209

[44] Duan G, Yao M, Ma Y, Zhang W Perinatal and background risk factors for childhood autism in central China. Psychiatry Res. 2014;220(1-2):410-417. doi:10.1016/j.psychres.2014.05.057 25085792

[45] Durkin MS, DuBois LA, Maenner MJ Inter-pregnancy intervals and the risk of autism spectrum disorder: results of a population-based study. J Autism Dev Disord. 2015;45(7):2056-2066. doi:10.1007/s10803-015-2368-y 25636677 PMC4474747

[46] Eriksson MA, Westerlund J, Anderlid BM, Gillberg C, Fernell E First-degree relatives of young children with autism spectrum disorders: some gender aspects. Res Dev Disabil. 2012;33(5):1642-1648. doi:10.1016/j.ridd.2012.03.025 22554810

[47] Hultman CM, Sparén P, Cnattingius S Perinatal risk factors for infantile autism. Epidemiology. 2002;13(4):417-423. doi:10.1097/00001648-200207000-00009 12094096

[48] Kissin DM, Zhang Y, Boulet SL, Association of assisted reproductive technology (ART) treatment and parental infertility diagnosis with autism in ART-conceived children. Hum Reprod. 2015;30(2):454-465. doi:10.1093/humrep/deu338 25518976 PMC4287306

[49] Glasson EJ, Bower C, Petterson B, de Klerk N, Chaney G, Hallmayer JF Perinatal factors and the development of autism: a population study. Arch Gen Psychiatry. 2004;61(6):618-627. doi:10.1001/archpsyc.61.6.618 15184241

[50] Zhang X, Lv CC, Tian J, Prenatal and perinatal risk factors for autism in China. J Autism Dev Disord. 2010;40(11):1311-1321. doi:10.1007/s10803-010-0992-0 20358271 PMC2974190

[51] El-Baz F, Ismael NA, El-Din SMN Risk factors for autism: an Egyptian study. Egypt J Med Hum Genet. 2011;12(1):31-38. doi:10.1016/j.ejmhg.2011.02.011

[52] Ji Y, Riley AW, Lee LC, Maternal biomarkers of acetaminophen use and offspring attention deficit hyperactivity disorder. Brain Sci. 2018;8(7):E127. doi:10.3390/brainsci8070127 29970852 PMC6071105

[53] Winkler-Schwartz A, Garfinkle J, Shevell MI Autism spectrum disorder in a term birth neonatal intensive care unit population. Pediatr Neurol. 2014;51(6):776-780. doi:10.1016/j.pediatrneurol.2014.07.009 25303867

[54] Al-Jammas IK, Al-Dobooni RM Prenatal and perinatal risk factors in autistic disorders. Arab J Psychiatry. 2012;23(2):108-114.

[55] Çak HT, Gökler B Attention deficit hyperactivity disorder and associated perinatal risk factors in preterm children. Turk Pediatri Ars. 2013;48(4):315-322. doi:10.4274/tpa.682

[56] Murray E, Pearson R, Fernandes M, Are fetal growth impairment and preterm birth causally related to child attention problems and ADHD? evidence from a comparison between high-income and middle-income cohorts. J Epidemiol Community Health. 2016;70(7):704-709. doi:10.1136/jech-2015-206222 26767410 PMC4941187

[57] Yeo J, Choi S, Joo Y, Kim H Prenatal, perinatal and developmental risk factors of attention-deficit hyperactivity disorder. J Korean Acad Child Adolesc Psychiatry. 2015;26(2):112-119. doi:10.5765/jkacap.2015.26.2.112

[58] Gustafsson P, Källén K Perinatal, maternal, and fetal characteristics of children diagnosed with attention-deficit-hyperactivity disorder: results from a population-based study utilizing the Swedish Medical Birth Register. Dev Med Child Neurol. 2011;53(3):263-268. doi:10.1111/j.1469-8749.2010.03820.x 20964677

[59] Silva D, Colvin L, Hagemann E, Bower C Environmental risk factors by gender associated with attention-deficit/hyperactivity disorder. Pediatrics. 2014;133(1):e14-e22. doi:10.1542/peds.2013-1434 24298003

[60] Sucksdorff M, Lehtonen L, Chudal R, Suominen A, Gissler M, Sourander A Lower Apgar scores and caesarean sections are related to attention-deficit/hyperactivity disorder. Acta Paediatr. 2018;107(10):1750-1758. doi:10.1111/apa.14349 29604108

[61] Ketzer CR, Gallois C, Martinez AL, Rohde LA, Schmitz M Is there an association between perinatal complications and attention-deficit/hyperactivity disorder-inattentive type in children and adolescents? Braz J Psychiatry. 2012;34(3):321-328. doi:10.1016/j.rbp.2012.01.001 23429778

[62] Halmøy A, Klungsøyr K, Skjærven R, Haavik J Pre- and perinatal risk factors in adults with attention-deficit/hyperactivity disorder. Biol Psychiatry. 2012;71(5):474-481. doi:10.1016/j.biopsych.2011.11.013 22200325

[63] Amiri S, Malek A, Sadegfard M, Abdi S Pregnancy-related maternal risk factors of attention-deficit hyperactivity disorder: a case-control study. ISRN Pediatr. 2012;2012:458064. doi:10.5402/2012/458064 22720167 PMC3374940

[64] Sussmann JE, McIntosh AM, Lawrie SM, Johnstone EC Obstetric complications and mild to moderate intellectual disability. Br J Psychiatry. 2009;194(3):224-228. doi:10.1192/bjp.bp.106.033134 19252150

[65] Bilder DA, Pinborough-Zimmerman J, Bakian AV, Prenatal and perinatal factors associated with intellectual disability. Am J Intellect Dev Disabil. 2013;118(2):156-176. doi:10.1352/1944-7558-118.2.156 23464612

[66] Leivonen S, Voutilainen A, Chudal R, Suominen A, Gissler M, Sourander A Obstetric and neonatal adversities, parity, and Tourette syndrome: a nationwide registry. J Pediatr. 2016;171:213-219. doi:10.1016/j.jpeds.2015.10.063 26608088

[67] Cubo E, Hortigüela M, Jorge-Roldan S, Prenatal and perinatal morbidity in children with tic disorders: a mainstream school-based population study in central Spain. Tremor Other Hyperkinet Mov (N Y). 2014;4:272. 25562036 10.7916/D8FN14W9 PMC4268040

[68] Cnattingius S, Hultman CM, Dahl M, Sparén P Very preterm birth, birth trauma, and the risk of anorexia nervosa among girls. Arch Gen Psychiatry. 1999;56(7):634-638. doi:10.1001/archpsyc.56.7.634 10401509

[69] Micali N, Kothari R, Nam KW, Eating disorder psychopathology, brain structure, neuropsychological correlates and risk mechanisms in very preterm young adults. Eur Eat Disord Rev. 2015;23(2):147-155. doi:10.1002/erv.2346 25645448

[70] Geller DA, Wieland N, Carey K, Perinatal factors affecting expression of obsessive compulsive disorder in children and adolescents. J Child Adolesc Psychopharmacol. 2008;18(4):373-379. 18759647 10.1089/cap.2007.0112 PMC2935829

[71] Vasconcelos MS, Sampaio AS, Hounie AG, Prenatal, perinatal, and postnatal risk factors in obsessive-compulsive disorder. Biol Psychiatry. 2007;61(3):301-307. doi:10.1016/j.biopsych.2006.07.014 17123475

[72] Hultman CM, Sparén P, Takei N, Murray RM, Cnattingius S Prenatal and perinatal risk factors for schizophrenia, affective psychosis, and reactive psychosis of early onset: case-control study. BMJ. 1999;318(7181):421-426. doi:10.1136/bmj.318.7181.421 9974454 PMC27730

[73] O’Neill SM, Curran EA, Dalman C, Birth by caesarean section and the risk of adult psychosis: a population-based cohort study. Schizophr Bull. 2016;42(3):633-641. doi:10.1093/schbul/sbv152 26615187 PMC4838084

[74] Bain M, Juszczak E, McInneny K, Kendell RE Obstetric complications and affective psychoses: two case-control studies based on structured obstetric records. Br J Psychiatry. 2000;176:523-526. doi:10.1192/bjp.176.6.523 10974956

[75] Chudal R, Sourander A, Polo-Kantola P, Perinatal factors and the risk of bipolar disorder in Finland. J Affect Disord. 2014;155:75-80. doi:10.1016/j.jad.2013.10.026 24215899 PMC3947252

[76] Gourion D, Arseneault L, Vitaro F, Brezo J, Turecki G, Tremblay RE Early environment and major depression in young adults: a longitudinal study. Psychiatry Res. 2008;161(2):170-176. doi:10.1016/j.psychres.2007.07.026 18849082

[77] Ordoñez AE, Bobb A, Greenstein D, Lack of evidence for elevated obstetric complications in childhood onset schizophrenia. Biol Psychiatry. 2005;58(1):10-15. doi:10.1016/j.biopsych.2005.02.009 15992518

[78] Karlsson H, Blomström Å, Wicks S, Yang S, Yolken RH, Dalman C Maternal antibodies to dietary antigens and risk for nonaffective psychosis in offspring. Am J Psychiatry. 2012;169(6):625-632. doi:10.1176/appi.ajp.2012.11081197 22535227

[79] Jones PB, Rantakallio P, Hartikainen AL, Isohanni M, Sipila P Schizophrenia as a long-term outcome of pregnancy, delivery, and perinatal complications: a 28-year follow-up of the 1966 north Finland general population birth cohort. Am J Psychiatry. 1998;155(3):355-364. doi:10.1176/ajp.155.3.355 9501745

[80] Harrison G, Fouskakis D, Rasmussen F, Tynelius P, Sipos A, Gunnell D Association between psychotic disorder and urban place of birth is not mediated by obstetric complications or childhood socio-economic position: a cohort study. Psychol Med. 2003;33(4):723-731. doi:10.1017/S0033291703007591 12785474

[81] Kendell RE, McInneny K, Juszczak E, Bain M Obstetric complications and schizophrenia: two case-control studies based on structured obstetric records. Br J Psychiatry. 2000;176:516-522. doi:10.1192/bjp.176.6.516 10974955

[82] Byrne M, Browne R, Mulryan N, Labour and delivery complications and schizophrenia: case-control study using contemporaneous labour ward records. Br J Psychiatry. 2000;176:531-536. doi:10.1192/bjp.176.6.531 10974958

[83] Hamadé A, Salameh P, Medlej-Hashim M, Hajj-Moussa E, Saadallah-Zeidan N, Rizk F Autism in children and correlates in Lebanon: a pilot case-control study. J Res Health Sci. 2013;13(2):119-124. 24077467

[84] Bilder D, Pinborough-Zimmerman J, Miller J, McMahon W Prenatal, perinatal, and neonatal factors associated with autism spectrum disorders. Pediatrics. 2009;123(5):1293-1300. doi:10.1542/peds.2008-0927 19403494

[85] Dalman C, Allebeck P, Cullberg J, Grunewald C, Köster M Obstetric complications and the risk of schizophrenia: a longitudinal study of a national birth cohort. Arch Gen Psychiatry. 1999;56(3):234-240. doi:10.1001/archpsyc.56.3.234 10078500

[86] Cannon M, Jones PB, Murray RM Obstetric complications and schizophrenia: historical and meta-analytic review. Am J Psychiatry. 2002;159(7):1080-1092. doi:10.1176/appi.ajp.159.7.1080 12091183

[87] Robson MS Classification of caesarean sections. Fetal Matern Med Rev. 2001;12(1):23-39. doi:10.1017/S0965539501000122

