# Supplementary material for: Association of Cesarean Delivery With Risk of Neurodevelopmental and Psychiatric Disorders in the Offspring: A Systematic Review and Meta-analysis
Source: JAMA Netw Open. 2019 Aug 28;2(8):e1910236. doi: 10.1001/jamanetworkopen.2019.10236 (PMC6716295; doi:10.1001/jamanetworkopen.2019.10236)
Supplement: Supplement. — eTable 1. Full search strategy eTable 2. Characteristics of the articles excluded due to duplicate data eTable 3. Variables adjusted for or matched by in each included study in the main analysis eTable 4. Leave-one-study-out analyses eTable 5. Subgroup analyses according to country’s income level and exposure ascertainment for neurodevelopmental and psychiatric disorders in caesarean-born offspring compared to vaginal-born offspring eTable 6. Meta-regression analyses eFigure 1. Publication bias: Funnel plots and Egger’s tests for the main analyses eFigure 2. Contour-enhanced funnel plot for ASD for potential publication bias eFigure 3. Forest Plots of the Results of Random Effects Meta-analyses Stratified by Type of Cesarean Delivery (Elective or Emergency) and the Disorders eFigure 4. Results of the association between birth by assisted vaginal delivery (compared to unassisted vaginal delivery) and neurodevelopmental and psychiatric disorders in the offspring eFigure 5. Sensitivity analyses: crude or least adjusted models eReferences [file jamanetwopen-2-e1910236-s001.pdf]

Supplementary Online Content

Zhang T, Sidorchuk A, Sevilla-Cermeño L, et al. Association of cesarean delivery with risk of neurodevelopmental and psychiatric disorders in the offspring: a systematic review and meta-analysis. *JAMA Netw Open*. 2019;2(8):e1910236. doi:10.1001/jamanetworkopen.2019.10236

eTable 1. Full search strategy

eTable 2. Characteristics of the articles excluded due to duplicate data

eTable 3. Variables adjusted for or matched by in each included study in the main analysis

eTable 4. Leave-one-study-out analyses

eTable 5. Subgroup analyses according to country's income level and exposure ascertainment for neurodevelopmental and psychiatric disorders in caesarean-born offspring compared to vaginal-born offspring

eTable 6. Meta-regression analyses

eFigure 1. Publication bias: Funnel plots and Egger's tests for the main analyses

eFigure 2. Contour-enhanced funnel plot for ASD for potential publication bias

eFigure 3. Forest Plots of the Results of Random Effects Meta-analyses Stratified by Type of Cesarean Delivery (*Elective* or *Emergency*) and the Disorders

eFigure 4. Results of the association between birth by assisted vaginal delivery (compared to *unassisted* vaginal delivery) and neurodevelopmental and psychiatric disorders in the offspring

eFigure 5. Sensitivity analyses: crude or least adjusted models

eReferences

This supplementary material has been provided by the authors to give readers additional information about their work.

© 2019 Zhang T et al. *JAMA Network Open.*

eTable 1. Full search strategy

Date: 19 December 2018
Topic/research question: Association of obstetric mode of delivery and psychiatric disorders in the offspring
Librarian(s): Magdalena Svanberg & GunBrit Knutssön (Karolinska Institutet University Library)

eTable 1a. Summary data for each database

Databases[a]	Number of hits
Medline (Ovid)	1911
Embase (embase.com)	2649
Web of Science Core Collection	1714
PsycINFO	679

[a] Full search strategy was presented for Medline database. Search strategy for other databases was similar to eTable 1b.

eTable 1b. Search terms for Medline search

#	Searches	Results
1	Delivery, Obstetric/	27303
2	exp Cesarean Section/	42113
3	exp Extraction, Obstetrical/	3306
4	Obstetrical Forceps/	1621
5	("c section*" or c-section* or cesar* or caesar* or delivery mode or delivery modes or elective cs or "mode of delivery" or obstetric* forcep* or planned cs* or ventouse*).ti,ab,kf.	63787
6	((obstetric* or vacuum) adj2 extract*).ti,ab,kf.	1931
7	((abdominal or assisted or forcep* or instrument* or operative or vacuum) adj2 (birth* or deliver* or vd)).ti,ab,kf.	7486
8	((birth or delivery or obstetric*) adj3 complication*).ti,ab,kf.	8687
9	(birth histor* or birth information).ti,ab,kf.	756
10	((obstetric* or peri-natal or perinatal) adj3 (characteristic* or factor* or risk*)).ti,ab,kf.	11138
11	or/1-10	112357
12	Mental Disorders/	153526
13	exp Anxiety Disorders/	75153
14	exp "Bipolar and Related Disorders"/	37869
15	exp "Disruptive, Impulse Control, and Conduct Disorders"/	7998
16	exp Dissociative Disorders/	4085
17	exp Elimination Disorders/	5264
18	exp "Feeding and Eating Disorders"/	28361
19	Mood Disorders/	13343
20	Depressive Disorder, Major/	26811
21	Depressive Disorder/	69223
22	Depressive Disorder, Treatment-Resistant/	906
23	Dysthymic Disorder/	1113
24	Cyclothymic Disorder/	653
25	exp Neurodevelopmental Disorders/	167979
26	Neurotic Disorders/	17943
27	exp "Schizophrenia Spectrum and Other Psychotic Disorders"/	140016
28	exp Substance-Related Disorders/	262328
29	exp "Trauma and Stressor Related Disorders"/	36310
**#**	**Searches**	**Results**

© 2019 Zhang T et al. *JAMA Network Open.*

30	(adhd or attention deficit or anorexia or asd or autism or autistic or binge eating or bipolar* or bulimia* or compulsive or depress* or dyscalculia* or dyslexia* or neurosis or neuroses or ocd or obsessive or panic or paranoi* or phobia or phobic or psychosis or psychoses or psychological trauma* or psychotic or ptsd or schizophren* or tics).ti,ab,kf.	747722
31	((acute stress or adjustment or anxiety or conduct or dissociative or disruptive or eating or hyperactivity or learning or mood or neurodevelopmental or neurotic or personality or posttrauma* or tic or traumatic stress) adj3 (disease* or disorder*)).ti,ab,kf.	145950
32	((development* or intellectual*) adj2 (disorder* or disabilit*)).ti,ab,kf.	31244
33	(mental* adj2 (disease* or disorder* or ill* or problem*)).ti,ab,kf.	90872
34	((alcohol or drug* or substance*) adj3 (abuse or disorder* or misuse)).ti,ab,kf.	90017
35	or/12-34	1361923
36	exp Case-Control Studies/	960041
37	Control Groups/	1601
38	Matched-Pair Analysis/	4661
39	exp Cohort studies/	1807116
40	Cross-Sectional Studies/	281284
41	observational study.pt.	55739
42	Registries/	77457
43	Prevalence/	261164
44	(cohort* or cross sectional or control group* or longitudinal or observational or prevalence or prospective or register stud* or registr* or retrospective or transversal).ti,ab,kf.	2576728
45	(case* adj5 control*).ti,ab,kf.	171126
46	(case adj3 comparison*).ti,ab,kf.	2894
47	or/36-46	3768258
48	exp Child/	1801194
49	Adolescent/	1901581
50	Infant/	750175
51	Siblings/	10077
52	Twin study/	8005
53	Prenatal Exposure Delayed Effects/	26420
54	(adoles* or adult* or boy* or child* or girl* or infant* or infancy or kid or kids or offspring or school age or schoolchild* or sibling* or teen* or toddler* or twin*).ti,ab,kf.	2855577
55	or/48-54	4503500
56	11 and 35 and 47 and 55	1911

© 2019 Zhang T et al. *JAMA Network Open.*

eTable 2. Characteristics of the articles excluded due to duplicate data

Study	Study location Period	Study design	Number of participants		Outcome(s)	Outcome assessments	Offspring' age range	Variables adjusted for	Matched factors	Newcastle-Ottawa score
			Caesarean section	Vaginal delivery						
Bilder, 2009[1]	United States 2002	Case-Control	1331	11068	ASD including Autism, PDD-NOS, and AS	ICD-9 and DSM-IV-TR	8 years old	Maternal age, gestational length, and parity	Gender and birth year	7
Brimacombe, 2007[2]	United States 2001-2003	Cohort	NR	NR	ASD including autism, PDD-NOS, and AS	DSM-IV, Autism Diagnostic Interview-Revised, Autism Diagnostic Observation Schedule-Generic, CARS	2 to 18 years	None	/	5
Dalman, 1999[3]	Sweden 1973-1995	Cohort	NR	NR	Schizophrenia	ICD-9 code 295	10-22 years	Psychotic illness in mother, maternal age, marital status, child's birth year, birth hospital and sex.	/	8

© 2019 Zhang T et al. *JAMA Network Open.*

Study	Study location Period	Study design	Number of participants		Outcome(s)	Outcome assessments	Offspring' age range	Variables adjusted for	Matched factors	Newcastle-Ottawa score
			Caesarean section	Vaginal delivery						
Gunnel, 2003[4]	Sweden 1973-1997	Cohort	20,558	226,097	Non-affective psychosis	ICD-9 codes 295, 297-299 and ICD-10 codes F20, F21-F29	Mean age at first hospital admission was 21 years	None	/	6
Nilsen, 2013[5]	Norway 1999-2010	Cohort	41,279	260,081	ASD, including childhood autism, atypical autism, AS, other PDD, and PDD, unspecified.	the Autism Diagnostic Interview - Revised, the Autism Diagnostic Observation Schedule, DSM-IV, and ICD-10 codes F84.0, F84.1, F84.5, F84.8, F84.9.	3-11 years	Year of birth, maternal age, paternal age, marital status, parity, and hospital size.	/	9

Abbreviations: ASD, autism spectrum disorders; PDD-NOS, pervasive developmental disorder-not otherwise specified; AS, Asperger's Syndrome; DSM, The Diagnostic and Statistical Manual of Mental Disorders; ICD, the International Statistical Classification of Diseases and Related Health Problems; CARS, Childhood Autism Rating Scale; NR, not reported.

© 2019 Zhang T et al. *JAMA Network Open.*

eTable 3. Variables adjusted for or matched by in each study included in the main analysis

Study	Adjusted for	Matched for
Al-Jammas, 2012[6]	/	/
Amiri, 2012[7]	/	/
Axelsson, 2018[8 a]	/	/
Axelsson, 2019[9 a]	/	/
Bain, 2000[10]	/	The obstetric unit of birth, gender, data of birth, maternal age, maternal parity and father's occupation.
Bilder, 2013[11]	/	/
Brander, 2016[12 a]	/	/
Brander, 2018[13 a]	/	/
Burstyn, 2010[14]	/	/
Byrne, 2000[15]	/	Gender, maternal age, parity, social class and home/hospital birth
Cak, 2013[16]	/	/
Chen, 2017[17 a]	/	/
Chien, 2015[18]	/	/
Chudal, 2014[19]	Maternal age, psychiatric history, education level, birth place, maternal smoking during pregnancy and number of pervious birth	Sex, date of birth and residence in Finland on the first date of diagnosis of the case
Cnattingius, 1999[20]	/	Year and hospital of birth
Cubo, 2014[21]	Family history of tics, BMI, and presence of any coexistent comorbid neuropsychiatric disturbances	Age and gender
Curran, 2015[22 a]	/	/
Curran, 2016[23 a]	/	/
Dodds, 2011[24]	/	
Duan, 2014[25]	Parental age, parents' level of education, parents' character, family history of psychiatric disorder, stress, passive smoking, pregnancy complications, premature delivery, jaundice, threatened abortion, infections	age and gender
Durkin, 2015[26]	/	/
El-Baz, 2011[27]	/	Age, gender, environment and habit

© 2019 Zhang T et al. *JAMA Network Open.*

Study	Adjusted for	Matched for
Eriksson, 2012[28]	/	/
Geller, 2008[29]	/	Age, gender, socioeconomic status, and family intact status.
Glasson, 2004[30]	/	Sex
Gourion, 2008[31]	Sex, perinatal condition, obstetrical complications, family adversity, maternal diagnostic of lifetime of major depressive disorder	/
Guisso, 2018[32]	Gender, age, delivery complications, and feeding difficulty, gestation variables, socioeconomic variables (including family history of psychiatric disease).	/
Gustafsson, 2011[33]	/	/
Haglund, 2011[34]	/	/
Halmoy, 2012[35]	Year of birth, maternal age, parity, marital status of mother, educational level of mother	/
Hamadé, 2013[36]	/	Age, region
Harrison, 2003[37]	/	/
Hultman, 1999[38]	/	Sex, year of birth, and hospital of birth
Hultman, 2002[39]	Maternal age, parity, smoking habits during pregnancy, mothers' country of birth, hypertensive diseases, diabetes, pregnancy bleeding, season of birth, gestational age, birth weight for gestational age, Apgar score at 5 minutes, congenital malformations	Sex, year, and hospital of birth
Hvelplund, 2016[40] [a]	/	/
Ji, 2018[41]	/	/
Jones, 1998[42]	Sex, socioeconomic status at birth, maternal Depression, and maternal smoking	/
Karlsson, 2012[43]	/	Sex, date of birth, birth hospital, and municipality.
Kendell, 2000[44]	/	the obstetric unit of birth, gender, date of birth (+2 months), maternal age (+2 years), maternal parity (first baby v. second or subsequent baby) and father's occupation (manual v. non-manual)
Ketzer, 2012[45]	IQ, maternal ADHD and cigarettes/day during pregnancy.	Gender and age
Kim, 2015[46]	Age, gender and socioeconomic status	/

© 2019 Zhang T et al. *JAMA Network Open.*

Study	Adjusted for	Matched for
Kissin, 2014[47]	/	/
Leivonen, 2016[48]	Maternal age, maternal SES, maternal psychiatric history, paternal age, and paternal psychiatric history.	Sex, date of birth, and place of birth
Maimburg, 2006[49]	Paternal age, mothers citizenship, birthweight and gestational age, Apgar, birth defect and irregular foetal position	Sex, year and country of birth
Maramara, 2014[50]	/	/
Matsuishi, 1999[51]	/	/
Micali, 2015[52]	Parental social class	/
Mrozek-Budzyn, 2013[53]	/	Year of birth, gender, and general practitioners
Murray, 2016[54]	/	/
O'Neill, 2016[55]	Gestational age, small for gestational age, Apgar score at 5min, maternal age, and maternal psychiatric history	/
Ordonez, 2005[56]	/	/
Polo-Kantola, 2014[57]	Maternal age, smoking during pregnancy, parity, and maternal psychiatric history.	Sex, date of birth, and place of birth
Razaz, 2018[58] [a]	/	/
Schieve, 2014[59]	/	Birth year, sex, maternal country of residence, race-ethnicity, age and education.
Silva, 2014[60]	Maternal age, Apgar at 5 min, year of birth, and socio-economic indexes for areas	Year of birth, gender, and socioeconomic status
Sucksdorff, 2018[61] [a]	/	Data of birth, sex and place of birth
Sussmann, 2009[62]	/	Age
Vasconcelos, 2007[63]	/	Gender and age
Winkler-Schwartz, 2014[64]	/	/
Yip, 2017[65]	Gestational age and paternal age	/
Zhang, 2010[66]	Paternal age at delivery, gender and birth year	Gender and birth year

[a] Studies originally adjusted for confounders but were re-calculated with 2 by 2 table in our main meta-analysis. Confounders that they originally adjusted for are:

© 2019 Zhang T et al. *JAMA Network Open.*

Axelsson, 2018: Childhood antibiotic use, parental age, parental education, maternal marital status, maternal smoking, infant sex, 5-min Apgar score, use of CPAP or a ventilator, asphyxia, parental epilepsy, preeclampsia or hypertension, gestational diabetes, parity, maternal antibiotic use during the pregnancy, maternal infection during the pregnancy, parental psychiatric history
Axelsson, 2019: Childhood antibiotic use, parental age, parental education, maternal marital status, maternal smoking, infant sex, 5-min Apgar score, use of CPAP or a ventilator, asphyxia, parental epilepsy, preeclampsia or hypertension, gestational diabetes, parity, maternal antibiotic use during the pregnancy, maternal infection during the pregnancy, parental psychiatric history
Brander, 2016: Sex, year of birth, age of mother and father, and parity.
Brander, 2018: Sex, year of birth, age of mother and father, and parity.
Chen, 2017: Gestational age, children's and parental characteristics
Curran, 2015: Year of birth, infant sex, maternal age, gestational age, 5-minute Apgar score, maternal and paternal country of birth, small for gestational age, large for gestational age, first born, family income, and maternal and paternal depression, bipolar disorder, and non-affective disorder.
Curran, 2016: Year of birth, infant gender, maternal age, maternal smoking during pregnancy, gestational age, 5-min Apgar score, maternal and paternal country of birth, small for gestational age, large for gestational age, firstborn, family income, maternal and paternal depression, bipolar disorder and non-affective disorder.
Hvelplund, 2016: Gender, gestational age, parents' ethnicity, parity, SGA, cesarean delivery, Apgar score <7 at 5 minutes, and congenital malformations.
Razaz, 2018: Maternal age, country of birth, education, mother cohabits with partner, parity, maternal height, smoking, year of delivery, gestational age at delivery, birth weight for gestational age
Sucksdorff, 2018: Gestational age and weight for gestational age, maternal age, psychiatric history, substance abuse, smoking, socioeconomic status and parity.

© 2019 Zhang T et al. *JAMA Network Open.*

eTable 4. Leave-one-study-out analyses

**Outcome**	**Study omitted**	**Pooled OR**	**95% CI**
Attention-deficit/hyperactivity disorder	Cak, 2013	1.17	(1.08, 1.27)
	Murray, 2016 (Pelotas)	1.18	(1.09, 1.28)
	Kim, 2015	1.17	(1.08, 1.27)
	Gustafsson, 2011	1.17	(1.08, 1.27)
	Axelsson, 2018	1.2	(1.10, 1.32)
	Silva, 2014	1.17	(1.08, 1.28)
	Curran, 2016	1.2	(1.07, 1.36)
	Sucksdorff, 2018	1.17	(1.06, 1.28)
	Chen, 2017	1.16	(1.07, 1.26)
	Halmoy, 2012	1.15	(1.05, 1.25)
	Ketzer, 2012	1.16	(1.07, 1.26)
	Ji, 2018	1.15	(1.06, 1.25)
	Murray, 2016 (ALSPAC)	1.16	(1.09, 1.28)
	Amiri, 2012	1.13	(1.08, 1.27)
	**Pooled estimate**	**1.17**	**(1.08, 1.27)**
Autism spectrum disorders	Ji, 2018	1.32	(1.25 , 1.40)
	Chien, 2015	1.34	(1.26 , 1.43)
	Curran, 2015	1.34	(1.25 , 1.44)
	Glasson, 2004	1.31	(1.24 , 1.39)
	Yip, 2017 (Norway)	1.32	(1.25 , 1.41)
	Matsuishi, 1999	1.33	(1.25 , 1.41)
	Haglund, 2011	1.33	(1.25 , 1.41)
	Winkler-Schwartz, 2014	1.33	(1.25 , 1.41)
	Axelsson, 2019	1.35	(1.28 , 1.42)
	Al-Jammas, 2012	1.32	(1.25 , 1.40)
	Burstyn, 2010	1.35	(1.27 , 1.43)
	Eriksson, 2012	1.32	(1.25 , 1.40)
	Chen, 2017	1.33	(1.25 , 1.41)
	Polo-Kantola, 2014	1.32	(1.24 , 1.40)
	Schieve, 2014	1.33	(1.25 , 1.41)
	Dodds, 2011	1.34	(1.26 , 1.42)
	Yip, 2017 (Finland)	1.33	(1.25 , 1.42)
	Hultman, 2002	1.32	(1.25 , 1.40)
	Duan, 2014	1.33	(1.25 , 1.41)
	Durkin, 2015	1.33	(1.25 , 1.41)
	Mrozek-Budzyn, 2013	1.33	(1.26 , 1.42)
	Maramara, 2014	1.34	(1.26 , 1.42)
	Zhang, 2010	1.33	(1.25 , 1.41)
	Yip, 2017 (WA)	1.31	(1.24 , 1.40)
	Kissin, 2014	1.31	(1.24 , 1.39)
	Hamadé, 2013	1.33	(1.26 , 1.41)
	Maimburg, 2006	1.33	(1.26 , 1.41)
	El-Baz, 2011	1.32	(1.25 , 1.40)
	Guisso, 2018	1.33	(1.25 , 1.41)

© 2019 Zhang T et al. *JAMA Network Open.*

	**Pooled estimate**	**1.33**	**(1.25. 1.41)**
**Outcome**	**Study omitted**	**Pooled OR**	**95% CI**
Intellectual disability	**Chen, 2017**	**2.61**	**(1.51, 4.53)**
	Sussmann, 2009	1.93	(0.82, 4.54)
	Bilder, 2013	1.26	(1.00, 1.60)
	**Pooled estimate**	**1.83**	**(0.90, 3.70)**
Tic disorder	Leivonen, 2016	2.4	(0.59, 9.81)
	Brander, 2017	2.21	(0.44, 11.16)
	**Cubo, 2014**	**1.24**	**(1.02, 1.50)**
	**Pooled estimate**	**1.31**	**(0.98, 1.76)**
Obsessive-compulsive disorder	Brander, 2016	1.95	(0.93, 4.11)
	Geller, 2008	1.66	(0.68, 4.02)
	**Vasconcelos, 2007**	**1.13**	**(1.08, 1.19)**
	**Pooled estimate**	**1.49**	**(0.87, 2.56)**
Eating disorder	Cnattingius, 1999	1.23	(0.96, 1.58)
	Razaz, 2018	1.25	(0.93, 1.68)
	Micali, 2015	1.20	(0.82, 1.74)
	Hvelplund, 2016	1.08	(0.98, 1.18)
	**Pooled estimate**	**1.18**	**(0.96, 1.47)**
Major depression and affective psychoses	Hultman, 1999	1.06	(0.99, 1.14)
	O'Neill, 2016	1.2	(0.97, 1.48)
	Bain, 2000	1.06	(0.98, 1.14)
	Chudal, 2014	1.04	(0.97, 1.13)
	Gourion, 2008	1.06	(0.98, 1.14)
	**Pooled estimate**	**1.06**	**(0.98, 1.14)**
Non-affective psychoses	Karlsson, 2012	1.02	(0.82, 1.27)
	Harrison, 2003	1.06	(0.86, 1.31)
	O'Neill, 2016	0.88	(0.60, 1.28)
	Ordonez, 2005	1	(0.81, 1.24)
	Jones, 1998	0.98	(0.79, 1.22)
	Kendell, 2000	0.96	(0.76, 1.21)
	Byrne, 2000	0.87	(0.69, 1.11)
	**Pooled estimate**	**0.97**	**(0.78, 1.21)**

© 2019 Zhang T et al. *JAMA Network Open.*

eTable 5. Subgroup analyses according to country's income level and exposure ascertainment for neurodevelopmental and psychiatric disorders in caesarean-born offspring compared to vaginal-born offspring

Outcome	Country's income level			Exposure ascertainment		
	High income	Middle income	P-value	Medical records	Self-report	P-value
	OR (95% CI)	OR (95% CI)		OR (95% CI)	OR (95% CI)	
ADHD	1.14 (1.06-1.22)**	1.27 (0.62-2.59)**	0.93	1.13 (1.05-1.21)**	1.71 (0.84-3.49)**	0.51
ASD	1.33 (1.24-1.42)*	1.37 (1.14-1.64)	0.60	1.34 (1.26-1.42)**	1.17 (0.89-1.53)	0.56
Psychosis	1.05 (0.92-1.20)*	..	NA	1.02 (0.91-1.14)	..	NA

Note: Results shown are odds ratios with 95% confidence intervals. P-values presented in the table are for group differences. Significant results are highlighted in bold.
Abbreviations: ADHD, attention-deficit/hyperactivity disorder; ASD, autism spectrum disorders; OR, odds ratio; CI, confidence interval
NA: calculation of the p value is not applicable.
* Study heterogeneity medium (50%<I^2<75%), p-value<0.05
** Study heterogeneity high (I^2>75%), p-value<0.05

© 2019 Zhang T et al. *JAMA Network Open.*

eTable 6. Meta-regression analyses

	Test of moderators				Variance accounted for	Test for residual heterogeneity			
	No. of studies	Regression coefficient	95% CI	p value		Q_{res} statistic	I^2_{res}	DF	p value
For ADHD									
Publication year	11	-0.03	-0.11 - 0.05	0.4	4.81%	53.09	83.05 %	9	<0.001
CS use proportion	11	0.69	-0.75 - 2.14	0.31	30.14%	62.62	85.63 %	9	<0.001
Study design	11	-0.19	-0.59 – 0.21	0.31	0.7%	51.05	82.37 %	9	<0.001
Country income	11	-0.78	-1.41 – (-0.16)	**0.02**	**77.3%**	47.24	80.95 %	9	<0.001
Exposure ascertainment	11	0.74	0.12-1.35	**0.02**	**76.48%**	47.72	81.14 %	9	<0.001
For ASD									
Publication year	28	-0.01	-0.03 - 0.003	0.11	23.49%	68.94	62.29 %	26	<0.001
CS use proportion	28	0.26	-0.33 - 0.85	0.38	-1.39%	87.81	70.39 %	26	<0.001
Study design	28	-0.11	-0.25 – 0.03	0.12	23.32%	71.63	63.70 %	26	<0.001
Country income	28	-0.11	-0.38 – 0.15	0.39	-0.03%	87.57	70.31 %	26	<0.001
Exposure ascertainment	27	-0.17	-0.35 – 0.10	0.26	-1.67%	86.81	71.2%	25	<0.001

Abbreviations: ADHD, attention-deficit/hyperactivity disorder; ASD, autism spectrum disorders; CI, confidence interval; DF: degree of freedom

© 2019 Zhang T et al. *JAMA Network Open.*

eFigure 1. Publication bias: Funnel plots and Egger's tests for the main analyses

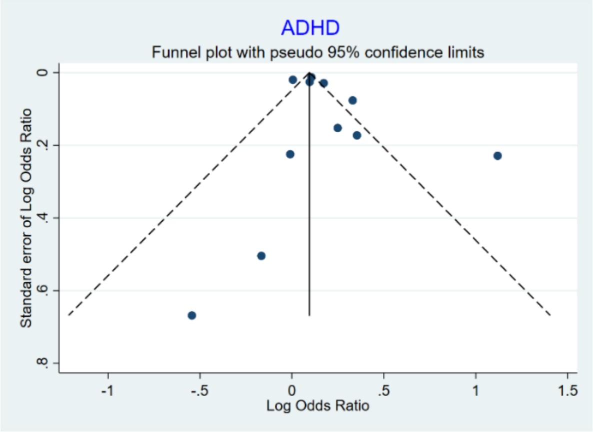

Egger's test for small-study effects:
Regress standard normal deviate of intervention
effect estimate against its standard error

Number of studies = 11 Root MSE = 2.455

Std_Eff	Coef.	Std. Err.	t	P>|t|	[95% Conf.	Interval]
slope	.0707799	.0308947	2.29	0.048	.0008913	.1406685
bias	1.180413	.9986772	1.18	0.268	-1.078752	3.439577

Test of H0: no small-study effects P = 0.268

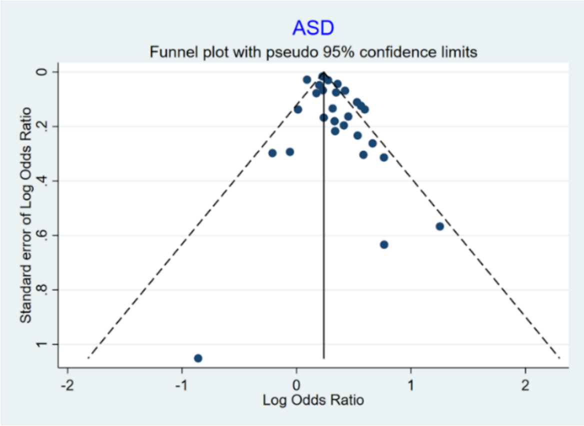

Egger's test for small-study effects:
Regress standard normal deviate of intervention
effect estimate against its standard error

Number of studies = 28 Root MSE = 1.699

Std_Eff	Coef.	Std. Err.	t	P>|t|	[95% Conf.	Interval]
slope	.2000437	.0251453	7.96	0.000	.1483567	.2517307
bias	.9799376	.4305486	2.28	0.031	.0949322	1.864943

Test of H0: no small-study effects P = 0.031

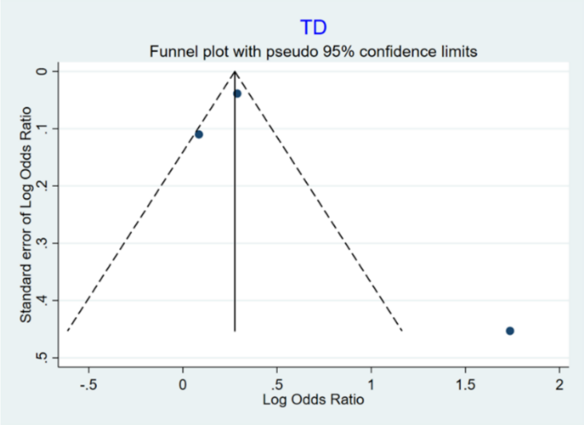

Egger's test for small-study effects:
Regress standard normal deviate of intervention
effect estimate against its standard error

Number of studies = 3 Root MSE = 3.268

Std_Eff	Coef.	Std. Err.	t	P>|t|	[95% Conf.	Interval]
slope	.1994783	.1895517	1.05	0.484	-2.209005	2.607961
bias	1.55448	3.014338	0.52	0.697	-36.74632	39.85527

Test of H0: no small-study effects P = 0.697

© 2019 Zhang T et al. *JAMA Network Open.*

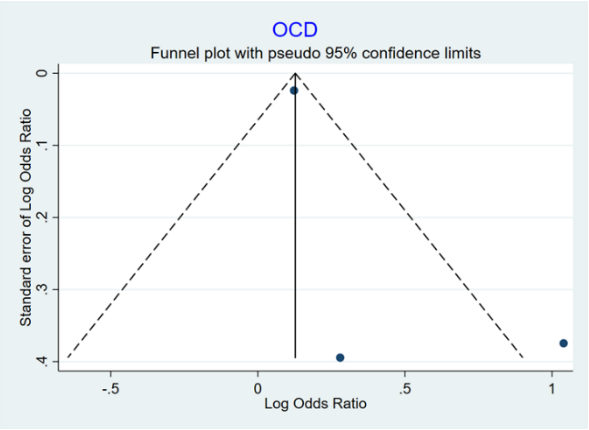


```
Egger's test for small-study effects:
Regress standard normal deviate of intervention
effect estimate against its standard error

Number of studies =   3                                     Root MSE      =   1.452
------------------------------------------------------------------------------
     Std_Eff |      Coef.   Std. Err.       t    P>|t|     [95% Conf. Interval]
-------------+----------------------------------------------------------------
       slope |     .08673   .0454468     1.91   0.307    -.4907261    .6641861
        bias |   1.514836   1.099458     1.38   0.400    -12.45511    15.48478
------------------------------------------------------------------------------

Test of H0: no small-study effects          P = 0.400
```

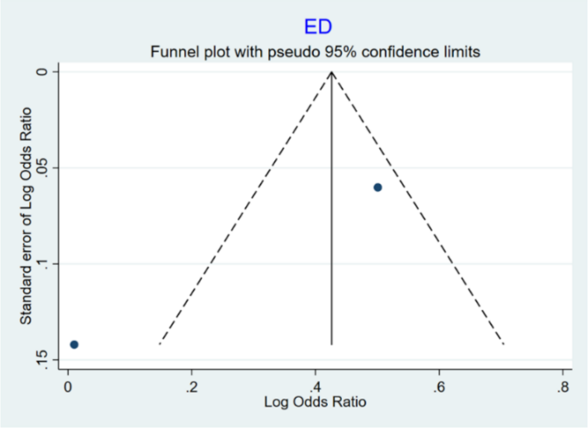

Egger's test was not performed since there were less than three studies were included.

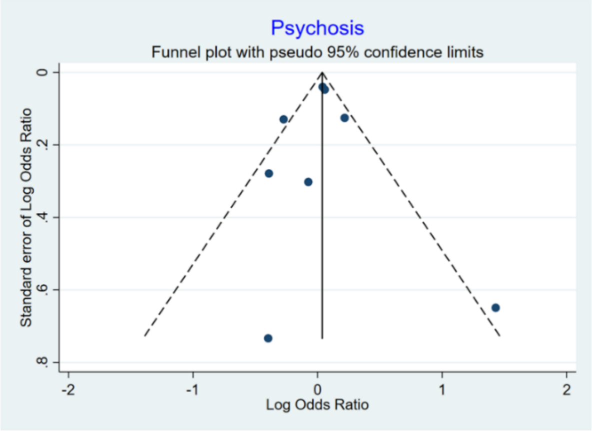


```
Egger's test for small-study effects:
Regress standard normal deviate of intervention
effect estimate against its standard error

Number of studies =   8                                     Root MSE      =   1.594
------------------------------------------------------------------------------
     Std_Eff |      Coef.   Std. Err.       t    P>|t|     [95% Conf. Interval]
-------------+----------------------------------------------------------------
       slope |   .0489981   .0661743     0.74   0.487    -.1129245    .2109208
        bias |   -.206496    .817432    -0.25   0.809     -2.20668    1.793688
------------------------------------------------------------------------------

Test of H0: no small-study effects          P = 0.809
```

© 2019 Zhang T et al. *JAMA Network Open.*

eFigure 2. Contour-enhanced funnel plot for ASD **for potential publication bias**

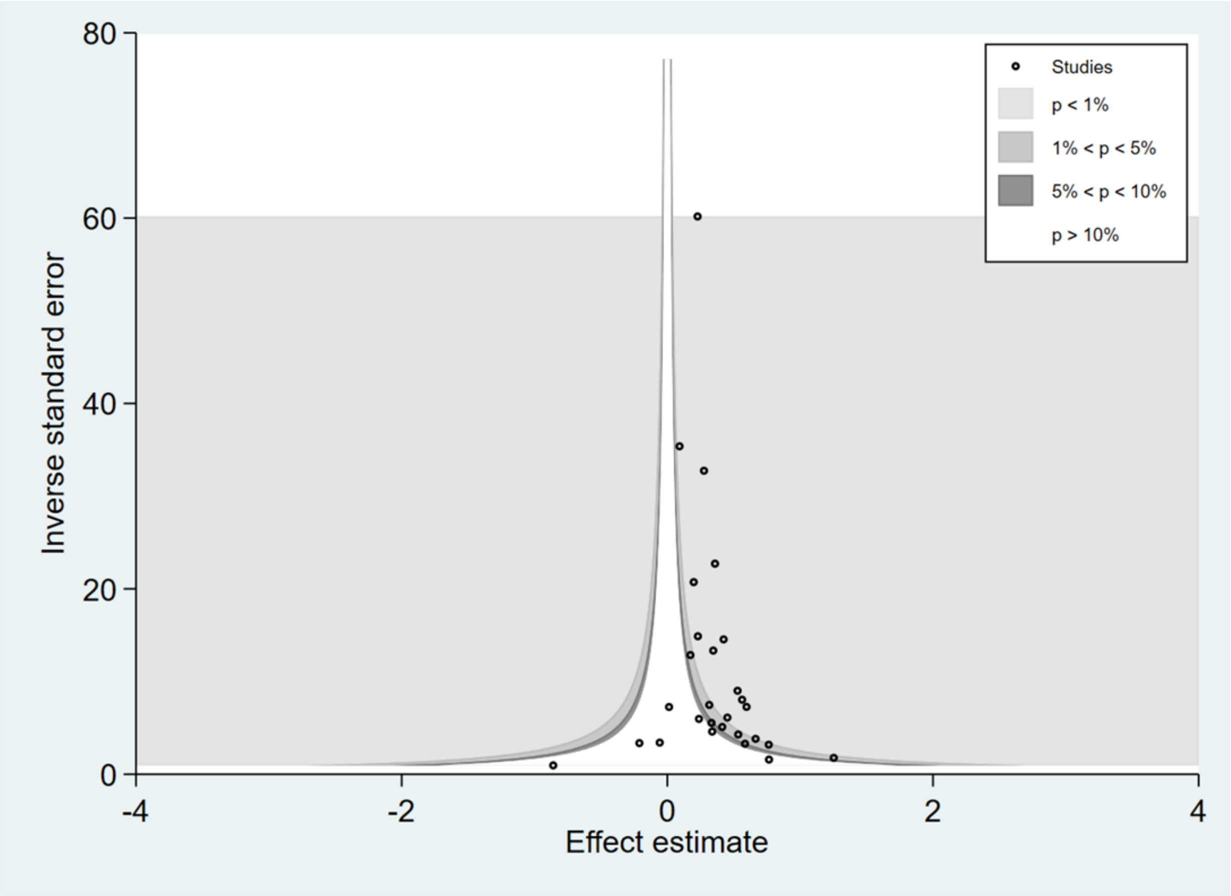

There is a suggestion of missing studies on the darker area (i.e. indicating high statistical significance) of left-hand-side of the plot, for which publication bias is a plausible explanation.

© 2019 Zhang T et al. *JAMA Network Open.*

eFigure 3. Forest Plots of the Results of Random Effects Meta-analyses Stratified by Type of Cesarean Delivery (Elective or Emergency) and the Disorders

A. *Elective* caesarean section compared to *unassisted* vaginal delivery

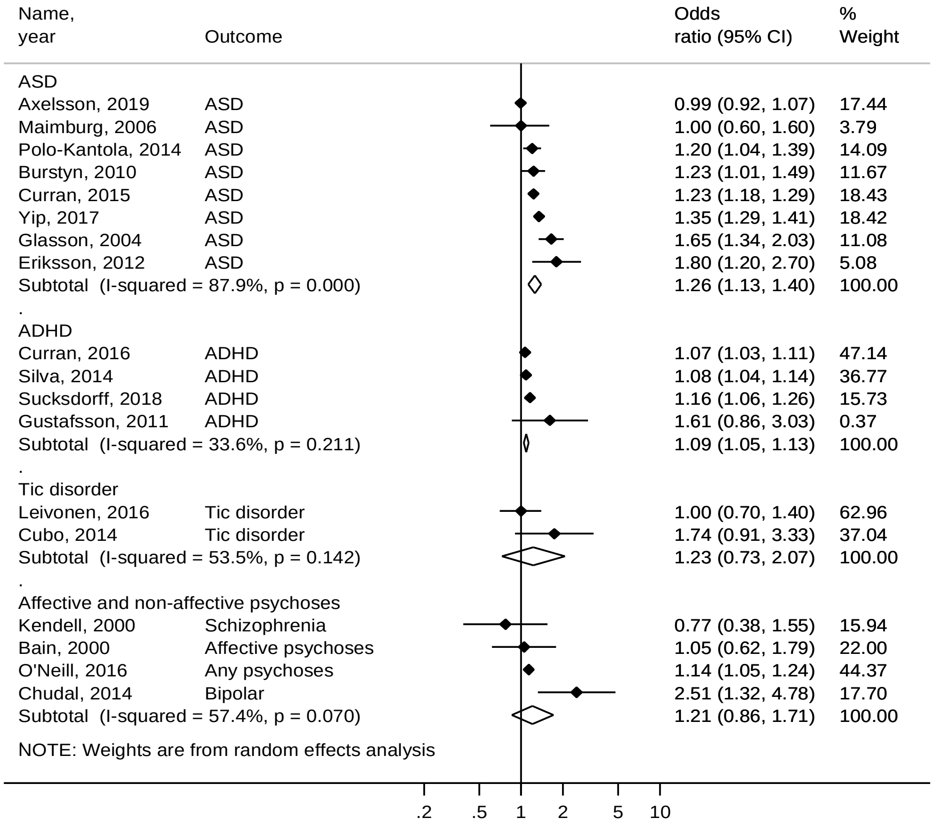

B. *Emergency* caesarean section compared to *unassisted* vaginal delivery

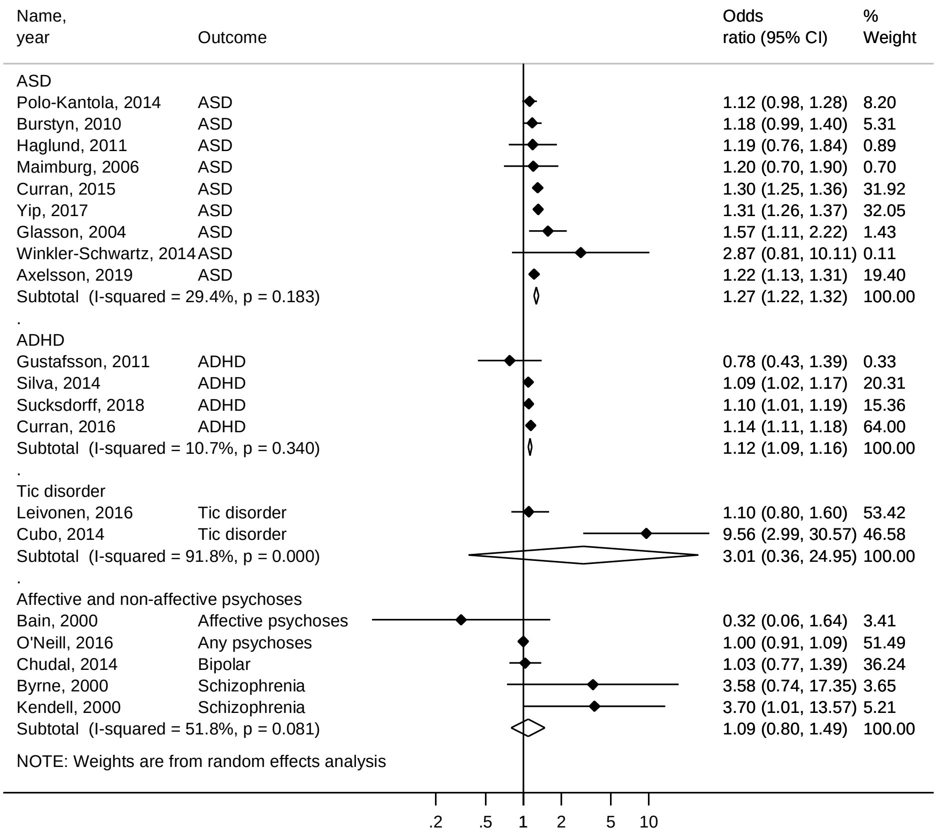

© 2019 Zhang T et al. *JAMA Network Open.*

eFigure 4. Results of the association between birth by *assisted* vaginal delivery (compared to *unassisted* vaginal delivery) and neurodevelopmental and psychiatric disorders in the offspring

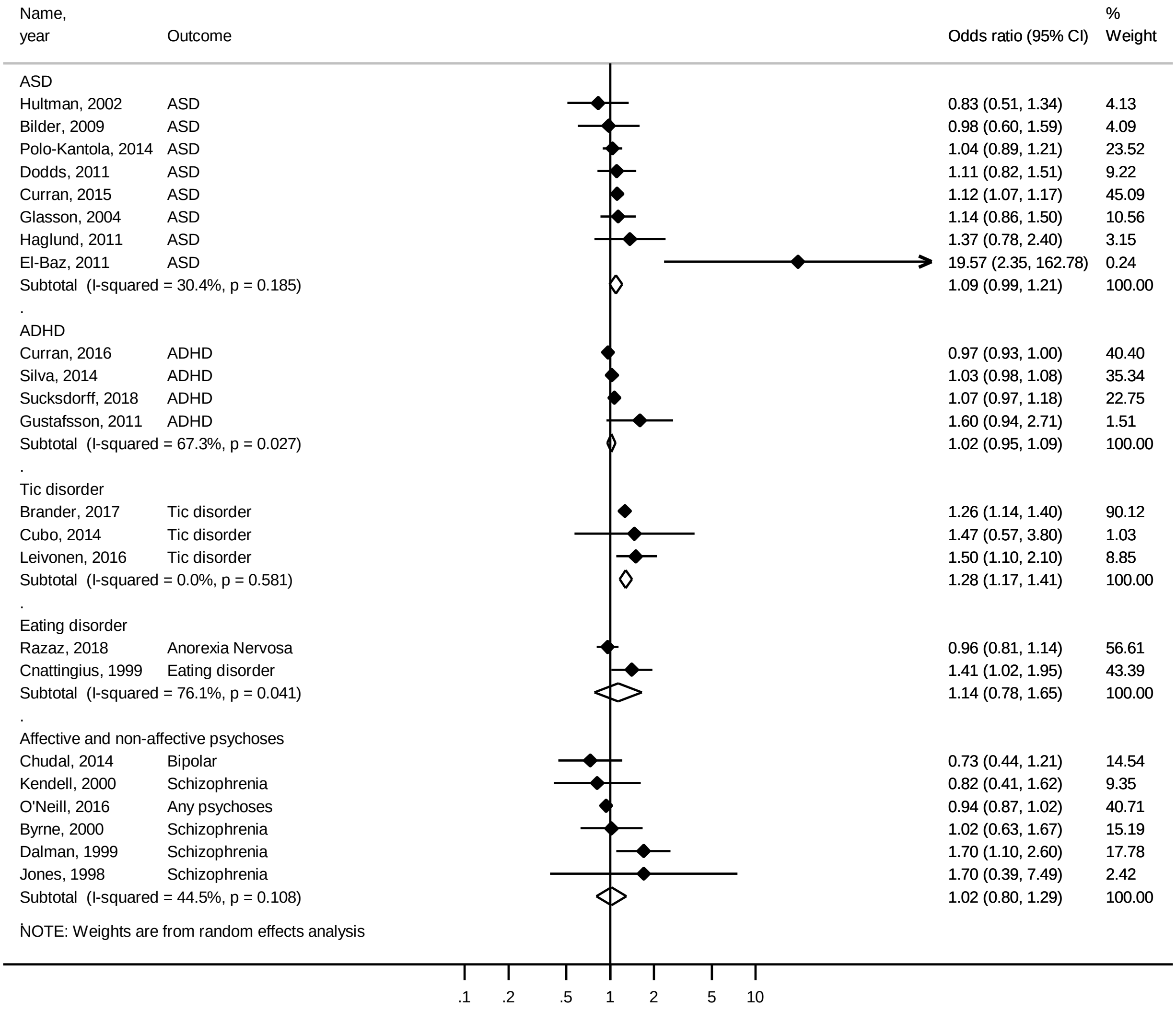

Abbreviations: ADHD: attention-deficit hyperactivity disorder; ASD: autism spectrum disorder

© 2019 Zhang T et al. *JAMA Network Open.*

eFigure 5. Sensitivity analysis**:** Crude or least adjusted models

eFigure 5a. Forest plot of crude or least adjusted estimates of the association between birth by *caesarean section* and neurodevelopmental and psychiatric disorders in the offspring

Name, year	Outcome	Caesarean section case/delivery	Vaginal delivery case/delivey	Odds ratio (95% CI)	% Weight
ASD					
Matsuishi, 1999	ASD	1/18	25/205	0.42 (0.05, 3.32)	0.08
Mrozek-Budzyn, 2013	ASD	21/74	67/204	0.81 (0.45, 1.45)	0.85
Hamadé, 2013	ASD	24/74	62/184	0.94 (0.53, 1.68)	0.88
Maramara, 2014	ASD	73/31872	190/84023	1.01 (0.77, 1.33)	2.92
Burstyn, 2010	ASD	304/49456	834/170572	1.26 (1.10, 1.44)	5.91
Axelsson, 2019	ASD	1564/119433	6703/560440	1.10 (1.04, 1.16)	8.01
Maimburg, 2006	ASD	76/633	371/4099	1.37 (1.05, 1.78)	3.04
Dodds, 2011	ASD	218/26754	706/102948	1.19 (1.02, 1.39)	5.33
Chien, 2015	ASD	684/174376	1166/362297	1.22 (1.11, 1.34)	6.99
Curran, 2015	ASD	4330/340108	23960/2357206	1.26 (1.22, 1.30)	8.44
Guisso, 2018	ASD	64/125	72/189	1.70 (1.08, 2.69)	1.31
Yip, 2017 (Finland)	ASD	1331/156863	5753/891684	1.32 (1.24, 1.40)	7.91
Schieve, 2014	ASD	NA	NA	1.38 (1.25, 1.52)	6.90
Haglund, 2011	ASD	36/7434	214/61530	1.39 (0.98, 1.99)	1.98
Chen, 2017	ASD	36/6285	52/12698	1.40 (0.91, 2.15)	1.47
Yip, 2017 (Norway)	ASD	213/127922	1090/924475	1.41 (1.22, 1.64)	5.48
Polo-Kantola, 2014	ASD	860/3349	3328/17117	1.43 (1.31, 1.56)	7.23
Duan, 2014	ASD	152/287	134/285	1.27 (0.91, 1.76)	2.22
Durkin, 2015	ASD	33/4624	127/26843	1.51 (1.03, 2.22)	1.74
Yip, 2017 (WA)	ASD	289/65953	802/279228	1.53 (1.34, 1.75)	5.82
Eriksson, 2012	ASD	52/23286	134/94242	1.57 (1.14, 2.17)	2.30
Hultman, 2002	ASD	87/352	321/2096	1.80 (1.40, 2.40)	2.93
Kissin, 2014	ASD	328/27152	109/15231	1.70 (1.36, 2.11)	3.81
Glasson, 2004	ASD	134/380	331/1398	1.76 (1.38, 2.24)	3.33
Zhang, 2010	ASD	43/77	43/104	1.79 (0.99, 3.25)	0.82
El-Baz, 2011	ASD	39/89	61/213	1.94 (1.16, 3.25)	1.07
Ji, 2018	ASD	22/214	22/434	2.15 (1.16, 3.97)	0.77
Winkler-Schwartz, 2014	ASD	7/35	5/48	2.15 (0.62, 7.45)	0.20
Al-Jammas, 2012	ASD	14/19	36/81	3.50 (1.15, 10.63)	0.25
Subtotal (I-squared = 69.7%, p = 0.000)				1.36 (1.28, 1.43)	100.00
.					
ADHD					
Cak, 2013	ADHD	18/80	4/12	0.58 (0.16, 2.15)	0.29
Murray, 2016 (Pelotas)	ADHD	NA	NA	0.79 (0.51, 1.20)	2.44
Kim, 2015	ADHD	24/34	34/46	0.85 (0.32, 2.28)	0.51
Gustafsson, 2011	ADHD	22/2996	215/29016	0.99 (0.64, 1.54)	2.32
Axelsson, 2018	ADHD	3167/117863	14804/553727	1.01 (0.97, 1.05)	17.73
Silva, 2014	ADHD	2819/8863	10363/34829	1.10 (1.05, 1.16)	17.13
Curran, 2016	ADHD	7230/238687	40548/1483861	1.11 (1.08, 1.14)	18.27
Sucksdorff, 2018	ADHD	1892/8034	8428/40963	1.19 (1.12, 1.26)	16.73
Chen, 2017	ADHD	71/6320	112/12758	1.28 (0.95, 1.73)	4.39
Ketzer, 2012	ADHD	NA	NA	1.30 (0.80, 2.00)	2.16
Halmoy, 2012	ADHD	186/69051	2137/1103345	1.39 (1.20, 1.62)	10.27
Ji, 2018	ADHD	75/267	113/525	1.42 (1.02, 2.00)	3.61
Murray, 2016 (ALSPAC)	ADHD	NA	NA	1.54 (0.94, 2.52)	1.90
Amiri, 2012	ADHD	103/162	61/168	3.06 (1.96, 4.80)	2.24
Subtotal (I-squared = 80.7%, p = 0.000)				1.17 (1.09, 1.25)	100.00
.					
Intellectual disability					
Chen, 2017	Learning disability	111/6360	179/12825	1.25 (0.99, 1.59)	40.56
Sussmann, 2009	Intellectual disability	10/16	39/74	1.50 (0.49, 4.54)	20.75
Bilder, 2013	Intellectual disability	52/2679	94/14387	3.01 (2.14, 4.23)	38.69
Subtotal (I-squared = 88.2%, p = 0.000)				1.83 (0.90, 3.70)	100.00
.					
Tic disorder					
Leivonen, 2016	Tic disorder	128/581	610/2961	1.09 (0.88, 1.35)	40.84
Brander, 2017	Tic disorder	788/336063	4591/2611439	1.33 (1.24, 1.44)	46.59
Cubo, 2014	Tic disorder	23/31	41/122	5.68 (2.34, 13.80)	12.57
Subtotal (I-squared = 85.2%, p = 0.001)				1.47 (1.02, 2.13)	100.00
.					
Eating disorder					
Razaz, 2018	Anorexia Nervosa	265/53807	2108/428768	1.00 (0.88, 1.14)	28.23
Cnattingius, 1999	Eating disorder	65/387	716/4299	1.01 (0.76, 1.33)	24.28
Micali, 2015	Eating disorder	NA	NA	1.10 (0.70, 1.70)	19.04
Hvelplund, 2016	Eating disorder	386/173937	979/727290	1.65 (1.47, 1.86)	28.45
Subtotal (I-squared = 91.4%, p = 0.000)				1.18 (0.85, 1.63)	100.00
.					
OCD					
Brander, 2016	OCD	1987/248840	15110/2137846	1.13 (1.08, 1.19)	49.51
Geller, 2008	OCD	36/47	94/132	1.32 (0.61, 2.87)	24.60
Vasconcelos, 2007	OCD	31/47	37/91	2.83 (1.36, 5.89)	25.89
Subtotal (I-squared = 67.3%, p = 0.047)				1.49 (0.87, 2.56)	100.00
.					
Major depression and affective psychoses					
Hultman, 1999	Affective psychosis	14/89	184/1099	0.93 (0.51, 1.68)	1.54
O'Neill, 2016	Affective psychosis	696/125356	6485/1215881	1.04 (0.96, 1.13)	87.82
Bain, 2000	Affective psychosis	NA	NA	1.17 (0.64, 2.14)	1.48
Chudal, 2014	Bipolar	122/320	594/1792	1.24 (0.97, 1.59)	8.91
Gourion, 2008	Major depression disorder	NA	NA	1.70 (0.40, 7.70)	0.25
Subtotal (I-squared = 0.0%, p = 0.646)				1.06 (0.98, 1.14)	100.00
.					
Non-affective psychoses					
Ordonez, 2005	Schizophrenia	NA	NA	0.39 (0.10, 1.20)	4.28
Karlsson, 2012	Non-affective psychoses	18/85	193/679	0.68 (0.39, 1.17)	14.54
Jones, 1998	Schizophrenia	2/47	68/1097	0.67 (0.16, 2.83)	3.30
Harrison, 2003	Non-affective psychoses	64/60110	889/635915	0.76 (0.59, 0.98)	26.48
O'Neill, 2016	Non-affective psychoses	495/125155	4535/1213931	1.06 (0.96, 1.16)	32.91
Kendell, 2000	Schizophrenia	NA	NA	1.09 (0.63, 1.90)	14.38
Byrne, 2000	Schizophrenia	12/15	407/832	4.18 (1.17, 14.91)	4.11
Subtotal (I-squared = 60.6%, p = 0.018)				0.91 (0.69, 1.20)	100.00

NOTE: Weights are from random effects analysis

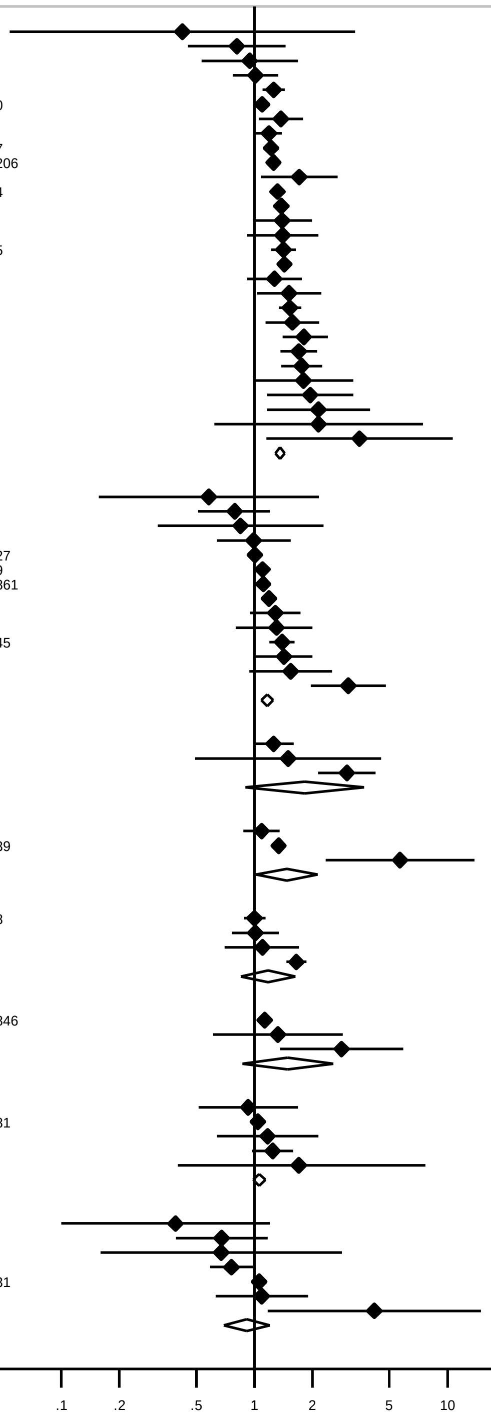

Abbreviations: NA: not available; ADHD: attention-deficit hyperactivity disorder; ASD: autism spectrum disorder; OCD: obsessive-compulsive disorder;

© 2019 Zhang T et al. *JAMA Network Open.*

eFigure 5b. Forest plot of crude or least adjusted estimates of the association between birth by *emergency CS* and neurodevelopmental and psychiatric disorders in the offspring

Name, year	Outcome	Odds ratio (95% CI)	% Weight
ASD			
Burstyn, 2010	ASD	1.18 (0.99, 1.40)	2.44
Haglund, 2011	ASD	1.19 (0.76, 1.84)	0.37
Maimburg, 2006	ASD	1.20 (0.70, 1.90)	0.29
Axelsson, 2019	ASD	1.22 (1.13, 1.31)	13.28
Curran, 2015	ASD	1.30 (1.25, 1.36)	38.51
Polo-Kantola, 2014	ASD	1.30 (1.16, 1.46)	5.50
Yip, 2017	ASD	1.31 (1.26, 1.37)	38.96
Glasson, 2004	ASD	1.57 (1.11, 2.22)	0.60
Winkler-Schwartz, 2014	ASD	2.87 (0.81, 10.11)	0.05
Subtotal (I-squared = 0.0%, p = 0.516)		1.29 (1.26, 1.33)	100.00
.			
ADHD			
Gustafsson, 2011	ADHD	0.78 (0.43, 1.39)	1.25
Silva, 2014	ADHD	1.09 (1.02, 1.17)	29.80
Curran, 2016	ADHD	1.14 (1.11, 1.18)	40.17
Sucksdorff, 2018	ADHD	1.25 (1.16, 1.35)	28.78
Subtotal (I-squared = 66.3%, p = 0.031)		1.15 (1.08, 1.23)	100.00
.			
Tic disorder			
Leivonen, 2016	Tic disorder	1.19 (0.90, 1.58)	53.82
Cubo, 2014	Tic disorder	9.56 (2.99, 30.57)	46.18
Subtotal (I-squared = 91.4%, p = 0.001)		3.12 (0.41, 23.84)	100.00
.			
Affective and non-affective psychoses			
Bain, 2000	Affective psychoses	0.32 (0.06, 1.64)	3.22
O'Neill, 2016	Any psychoses	1.00 (0.91, 1.09)	50.91
Chudal, 2014	Bipolar	1.11 (0.85, 1.45)	37.51
Byrne, 2000	Schizophrenia	3.58 (0.74, 17.35)	3.45
Kendell, 2000	Schizophrenia	3.70 (1.01, 13.57)	4.92
Subtotal (I-squared = 54.5%, p = 0.067)		1.12 (0.82, 1.51)	100.00

NOTE: Weights are from random effects analysis

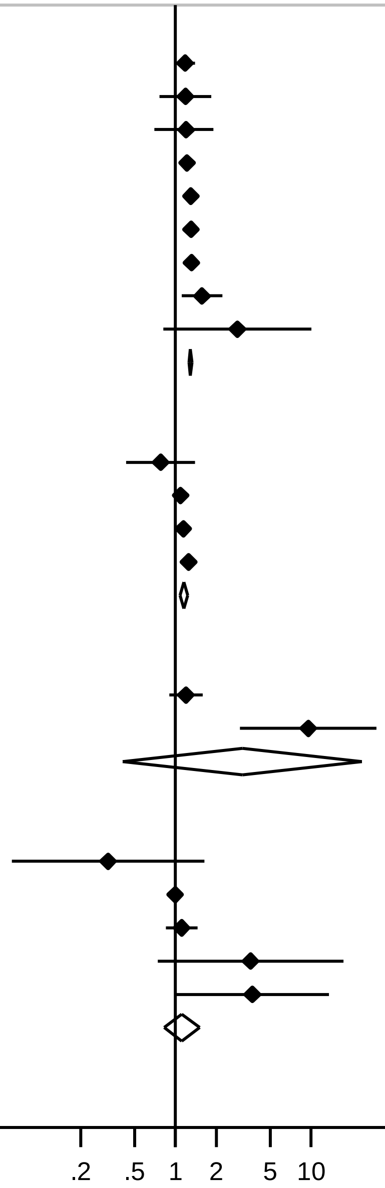

Abbreviations: NA: not available; ADHD: attention-deficit hyperactivity disorder; ASD: autism spectrum disorder

© 2019 Zhang T et al. *JAMA Network Open.*

eFigure 5c. Forest plot of crude or least adjusted estimates of the association between birth by elective CS and psychiatric disorders in the offspring

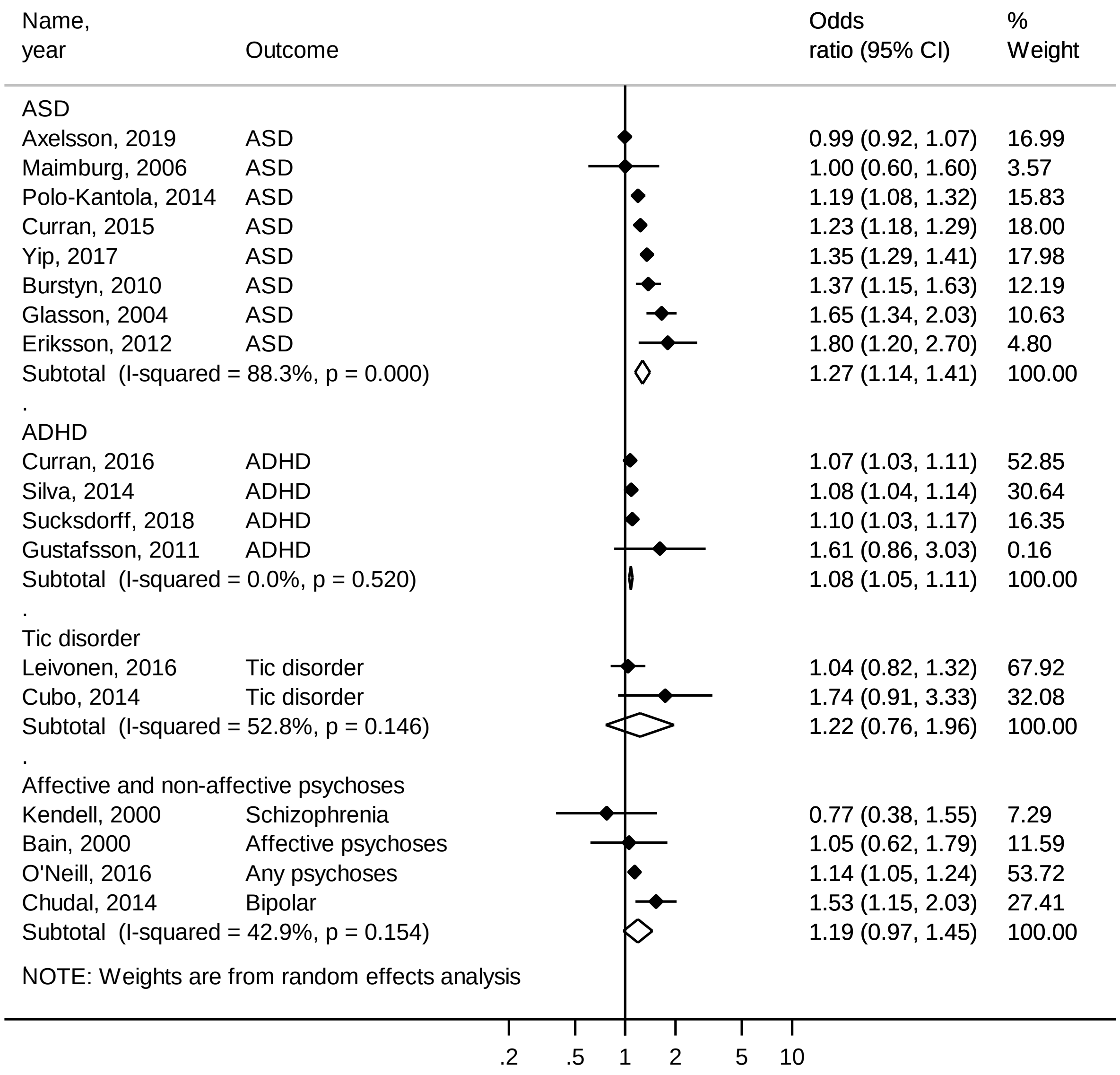

Abbreviations: NA: not available; ADHD: attention-deficit hyperactivity disorder; ASD: autism spectrum disorder

© 2019 Zhang T et al. *JAMA Network Open.*

eFigure 5d. Forest plot of crude or least adjusted estimates of the association between birth by *assisted* VD and psychiatric disorders in the offspring

Name, year	Outcome	Odds ratio (95% CI)	% Weight
ASD			
Hultman, 2002	ASD	0.83 (0.51, 1.34)	3.86
Bilder, 2009	ASD	0.98 (0.60, 1.59)	3.83
Dodds, 2011	ASD	1.11 (0.82, 1.51)	8.71
Curran, 2015	ASD	1.12 (1.07, 1.17)	45.65
Glasson, 2004	ASD	1.14 (0.86, 1.50)	10.00
Polo-Kantola, 2014	ASD	1.19 (1.03, 1.37)	24.79
Haglund, 2011	ASD	1.37 (0.78, 2.40)	2.94
El-Baz, 2011	ASD	19.57 (2.35, 162.78)	0.22
Subtotal (I-squared = 30.1%, p = 0.188)		1.13 (1.02, 1.25)	100.00
.			
ADHD			
Curran, 2016	ADHD	0.97 (0.93, 1.00)	40.24
Silva, 2014	ADHD	1.03 (0.98, 1.08)	34.76
Sucksdorff, 2018	ADHD	1.06 (0.97, 1.16)	23.63
Gustafsson, 2011	ADHD	1.60 (0.94, 2.71)	1.37
Subtotal (I-squared = 65.7%, p = 0.033)		1.02 (0.95, 1.08)	100.00
.			
Tic disorder			
Brander, 2017	Tic disorder	1.26 (1.14, 1.40)	88.98
Leivonen, 2016	Tic disorder	1.41 (1.04, 1.90)	10.00
Cubo, 2014	Tic disorder	1.47 (0.57, 3.80)	1.02
Subtotal (I-squared = 0.0%, p = 0.766)		1.28 (1.16, 1.41)	100.00
.			
Eating disorder			
Razaz, 2018	Anorexia Nervosa	0.96 (0.81, 1.14)	56.61
Cnattingius, 1999	Eating disorder	1.41 (1.02, 1.95)	43.39
Subtotal (I-squared = 76.1%, p = 0.041)		1.14 (0.78, 1.65)	100.00
.			
Affective and non-affective psychoses			
Chudal, 2014	Bipolar	0.79 (0.49, 1.25)	15.42
Kendell, 2000	Schizophrenia	0.82 (0.41, 1.62)	8.70
O'Neill, 2016	Any psychoses	0.94 (0.87, 1.02)	42.24
Byrne, 2000	Schizophrenia	1.02 (0.63, 1.67)	14.41
Dalman, 1999	Schizophrenia	1.70 (1.10, 2.60)	17.02
Jones, 1998	Schizophrenia	1.70 (0.39, 7.49)	2.21
Subtotal (I-squared = 42.0%, p = 0.125)		1.02 (0.82, 1.28)	100.00

NOTE: Weights are from random effects analysis

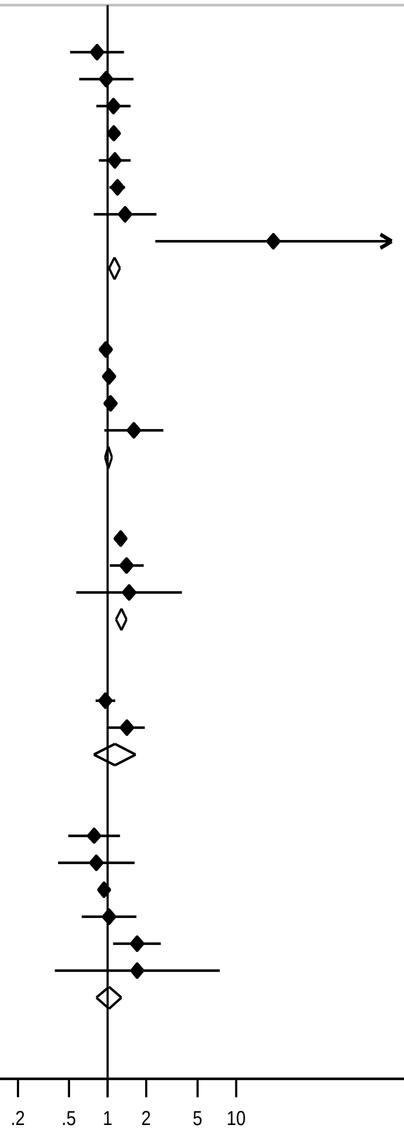

Abbreviations: NA: not available; ADHD: attention-deficit hyperactivity disorder; ASD: autism spectrum disorder

© 2019 Zhang T et al. *JAMA Network Open.*

eReferences

1. Bilder D, Pinborough-Zimmerman J, Miller J, McMahon W. Prenatal, perinatal, and neonatal factors associated with autism spectrum disorders. Pediatrics. 2009;123(5):1293-1300.
2. Brimacombe M, Ming X, Lamendola M. Prenatal and birth complications in autism. Matern Child Health J. 2007;11(1):73-79.
3. Dalman C, Allebeck P, Cullberg J, Grunewald C, Koster M. Obstetric complications and the risk of schizophrenia: a longitudinal study of a national birth cohort. Arch Gen Psychiatry. 1999;56(3):234-240.
4. Gunnell D, Rasmussen F, Fouskakis D, Tynelius P, Harrison G. Patterns of fetal and childhood growth and the development of psychosis in young males: A cohort study. Am J Epidemiol. 2003;158(4):291-300.
5. Nilsen RM, Suren P, Gunnes N, et al. Analysis of self-selection bias in a population-based cohort study of autism spectrum disorders. Paediatr Perinat Epidemiol. 2013;27(6):553-563.
6. Al-Jammas IK, Al-Dobooni RM. Prenatal and perinatal risk factors in autistic disorders. Arab Journal of Psychiatry. 2012;23(2):108-114.
7. Amiri S, Malek A, Sadegfard M, Abdi S. Pregnancy-related maternal risk factors of attention-deficit hyperactivity disorder: a case-control study. ISRN pediatr. 2012;2012:458064.
8. Axelsson PB, Clausen TD, Petersen AH, et al. Investigating the effects of cesarean delivery and antibiotic use in early childhood on risk of later attention deficit hyperactivity disorder. J Child Psychol Psychiatry. 2018;23:23.
9. Axelsson PB, Clausen TD, Petersen AH, et al. Relation Between Infant Microbiota and Autism?: Results from a National Cohort Sibling Design Study. Epidemiology. 2019;30(1):52-60.
10. Bain M, Juszczak E, McInneny K, Kendell RE. Obstetric complications and affective psychoses. Two case-control studies based on structured obstetric records. Br J Psychiatry. 2000;176:523-526.
11. Bilder DA, Pinborough-Zimmerman J, Bakian AV, et al. Prenatal and perinatal factors associated with intellectual disability. Am J Intellect Dev Disabil. 2013;118(2):156-176.
12. Brander G, Rydell M, Kuja-Halkola R, et al. Association of Perinatal Risk Factors With Obsessive-Compulsive Disorder A Population-Based Birth Cohort, Sibling Control Study. JAMA Psychiatry. 2016;73(11):1135-1144.
13. Brander G, Rydell M, Kuja-Halkola R, et al. Perinatal risk factors in Tourette's and chronic tic disorders: a total population sibling comparison study. Mol Psychiatry. 2018;23(5):1189-1197.
14. Burstyn I, Sithole F, Zwaigenbaum L. Autism spectrum disorders, maternal characteristics and obstetric complications among singletons born in Alberta, Canada. Chronic Dis Can. 2010;30(4):125-134.
15. Byrne M, Browne R, Mulryan N, et al. Labour and delivery complications and schizophrenia. Case-control study using contemporaneous labour ward records. Br J Psychiatry. 2000;176:531-536.
16. Çak HT, Gökler B. Attention deficit hyperactivity disorder and associated perinatal risk factors in preterm children. Turk Pediatri Arsivi. 2013;48(4):315-322.
17. Chen G, Chiang WL, Shu BC, et al. Associations of caesarean delivery and the occurrence of neurodevelopmental disorders, asthma or obesity in childhood based on Taiwan birth cohort study. BMJ Open. 2017;7(9):e017086.
18. Chien L-N, Lin H-C, Shao Y-HJ, Chiou S-T, Chiou H-Y. Risk of autism associated with general anesthesia during Cesarean delivery: A population-based birth-cohort analysis. J Autism Dev Disord. 2015;45(4):932-942.
19. Chudal R, Sourander A, Polo-Kantola P, et al. Perinatal factors and the risk of bipolar disorder in Finland. J Affect Disord. 2014;155:75-80.
20. Cnattingius S, Hultman CM, Dahl M, Sparen P. Very preterm birth, birth trauma, and the risk of anorexia nervosa among girls. Arch Gen Psychiatry. 1999;56(7):634-638.
21. Cubo E, Hortiguela M, Jorge-Roldan S, et al. Prenatal and Perinatal Morbidity in Children with Tic Disorders: A Mainstream School-based Population Study in Central Spain. Tremor Other Hyperkinet Mov (N Y). 2014;4:272.
22. Curran EA, Dalman C, Kearney PM, et al. Association Between Obstetric Mode of Delivery and Autism Spectrum Disorder: A Population-Based Sibling Design Study. JAMA Psychiatry. 2015;72(9):935-942.
23. Curran EA, Khashan AS, Dalman C, et al. Obstetric mode of delivery and attention-deficit/hyperactivity disorder: a sibling-matched study. Int J Epidemiol. 2016;45(2):532-542.
24. Dodds L, Fell DB, Shea S, et al. The role of prenatal, obstetric and neonatal factors in the development of autism. J Autism Dev Disord. 2011;41(7):891-902.
25. Duan G, Yao M, Ma Y, Zhang W. Perinatal and background risk factors for childhood autism in central China. Psychiatry Res. 2014;220(1-2):410-417.

© 2019 Zhang T et al. *JAMA Network Open.*

26. Durkin MS, DuBois LA, Maenner MJ. Inter-Pregnancy Intervals and the Risk of Autism Spectrum Disorder: Results of a Population-Based Study. J Autism Dev Disord. 2015;45(7):2056-2066.
27. El-Baz F, Ismael NA, El-Din SMN. Risk factors for autism: An Egyptian study. Egypt J Med Hum Genet. 2011;12(1):31-38.
28. Eriksson MA, Westerlund J, Anderlid BM, Gillberg C, Fernell E. First-degree relatives of young children with autism spectrum disorders: some gender aspects. Res Dev Disabil. 2012;33(5):1642-1648.
29. Geller DA, Wieland N, Carey K, et al. Perinatal factors affecting expression of obsessive compulsive disorder in children and adolescents. J Child Adolesc Psychopharmacol. 2008;18(4):373-379.
30. Glasson EJ, Bower C, Petterson B, et al. Perinatal factors and the development of autism: a population study. Arch Gen Psychiatry. 2004;61(6):618-627.
31. Gourion D, Arseneault L, Vitaro F, et al. Early environment and major depression in young adults: a longitudinal study. Psychiatry Res. 2008;161(2):170-176.
32. Guisso DR, Saadeh FS, Saab D, et al. Association of Autism with Maternal Infections, Perinatal and Other Risk Factors: A Case-Control Study. J Autism Dev Disord. 2018;48(6):2010-2021.
33. Gustafsson P, Källén K. Perinatal, maternal, and fetal characteristics of children diagnosed with attention-deficit-hyperactivity disorder: Results from a population-based study utilizing the Swedish Medical Birth Register. Dev Med Child Neurol. 2011;53(3):263-268.
34. Haglund NG, Kallen KB. Risk factors for autism and Asperger syndrome. Perinatal factors and migration. Autism. 2011;15(2):163-183.
35. Halmoy A, Klungsoyr K, Skjaerven R, Haavik J. Pre- and perinatal risk factors in adults with attention-deficit/hyperactivity disorder. Biol Psychiatry. 2012;71(5):474-481.
36. Hamade A, Salameh P, Medlej-Hashim M, et al. Autism in children and correlates in Lebanon: a pilot case-control study. J. 2013;13(2):119-124.
37. Harrison G, Fouskakis D, Rasmussen F, et al. Association between psychotic disorder and urban place of birth is not mediated by obstetric complications or childhood socio-economic position: a cohort study. Psychol Med. 2003;33(4):723-731.
38. Hultman CM, Sparen P, Takei N, Murray RM, Cnattingius S. Prenatal and perinatal risk factors for schizophrenia, affective psychosis, and reactive psychosis of early onset: case-control study. BMJ. 1999;318(7181):421-426.
39. Hultman CM, Sparen P, Cnattingius S. Perinatal risk factors for infantile autism. Epidemiology. 2002;13(4):417-423.
40. Hvelplund C, Hansen BM, Koch SV, Andersson M, Skovgaard AM. Perinatal Risk Factors for Feeding and Eating Disorders in Children Aged 0 to 3 Years. Pediatrics. 2016;137(2):e20152575.
41. Ji Y, Riley AW, Lee LC, et al. Maternal biomarkers of acetaminophen use and offspring attention deficit hyperactivity disorder. Brain Sci. 2018;8(7):E127.
42. Jones PB, Rantakallio P, Hartikainen AL, Isohanni M, Sipila P. Schizophrenia as a long-term outcome of pregnancy, delivery, and perinatal complications: a 28-year follow-up of the 1966 north Finland general population birth cohort. Am J Psychiatry. 1998;155(3):355-364.
43. Karlsson H, Blomstrom A, Wicks S, et al. Maternal antibodies to dietary antigens and risk for nonaffective psychosis in offspring. Am J Psychiatry. 2012;169(6):625-632.
44. Kendell RE, McInneny K, Juszczak E, Bain M. Obstetric complications and schizophrenia. Two case-control studies based on structured obstetric records. Br J Psychiatry. 2000;176:516-522.
45. Ketzer CR, Gallois C, Martinez AL, Rohde LA, Schmitz M. Is there an association between perinatal complications and attention-deficit/hyperactivity disorder-inattentive type in children and adolescents? Braz J Psychiatry. 2012;34(3):321-328.
46. Yeo J, Choi S, Joo Y, Kim H. Prenatal, perinatal and developmental risk factors of attention-deficit hyperactivity disorder. J Korean Acad Child Adolesc Psychiatry. 2015;26(2):112-119.
47. Kissin DM, Zhang Y, Boulet SL, et al. Association of assisted reproductive technology (ART) treatment and parental infertility diagnosis with autism in ART-conceived children. Hum Reprod. 2015;30(2):454-465.
48. Leivonen S, Voutilainen A, Chudal R, et al. Obstetric and Neonatal Adversities, Parity, and Tourette Syndrome: A Nationwide Registry. J Pediatr. 2016;171:213-219.
49. Maimburg RD, Vaeth M. Perinatal risk factors and infantile autism. Acta Psychiatr Scand. 2006;114(4):257-264.
50. Maramara LA, He W, Ming X. Pre- and perinatal risk factors for autism spectrum disorder in a New Jersey cohort. J Child Neurol. 2014;29(12):1645-1651.

© 2019 Zhang T et al. *JAMA Network Open.*

51. Matsuishi T, Yamashita Y, Ohtani Y, et al. Brief report: incidence of and risk factors for autistic disorder in neonatal intensive care unit survivors. J Autism Dev Disord. 1999;29(2):161-166.
52. Micali N, Kothari R, Nam KW, et al. Eating disorder psychopathology, brain structure, neuropsychological correlates and risk mechanisms in very preterm young adults. Eur Eat Disord Rev. 2015;23(2):147-155.
53. Mrozek-Budzyn D, Majewska R, Kieltyka A. Prenatal, perinatal and neonatal risk factors for autism - Study in Poland. Cent Eur J Med. 2013;8(4):424-430.
54. Murray E, Pearson R, Fernandes M, et al. Are fetal growth impairment and preterm birth causally related to child attention problems and ADHD? Evidence from a comparison between high-income and middle-income cohorts. J Epidemiol Community Health. 2016;70(7):704-709.
55. O'Neill SM, Curran EA, Dalman C, et al. Birth by Caesarean Section and the Risk of Adult Psychosis: A Population-Based Cohort Study. Schizophr Bull. 2016;42(3):633-641.
56. Ordonez AE, Bobb A, Greenstein D, et al. Lack of evidence for elevated obstetric complications in childhood onset schizophrenia. Biol Psychiatry. 2005;58(1):10-15.
57. Polo-Kantola P, Lampi KM, Hinkka-Yli-Salomaki S, et al. Obstetric risk factors and autism spectrum disorders in Finland. J Pediatr. 2014;164(2):358-365.
58. Razaz N, Cnattingius S. Association between maternal body mass index in early pregnancy and anorexia nervosa in daughters. Int J Eat Disord. 2018;51(8):906-913.
59. Schieve LA, Tian LH, Baio J, et al. Population attributable fractions for three perinatal risk factors for autism spectrum disorders, 2002 and 2008 autism and developmental disabilities monitoring network. Ann Epidemiol. 2014;24(4):260-266.
60. Silva D, Colvin L, Hagemann E, Bower C. Environmental risk factors by gender associated with attention-deficit/hyperactivity disorder. Pediatrics. 2014;133(1):e14-22.
61. Sucksdorff M, Lehtonen L, Chudal R, et al. Lower Apgar scores and Caesarean sections are related to attention-deficit/hyperactivity disorder. Acta Paediatr. 2018;107(10):1750-1758.
62. Sussmann JE, McIntosh AM, Lawrie SM, Johnstone EC. Obstetric complications and mild to moderate intellectual disability. Br J Psychiatry. 2009;194(3):224-228.
63. Vasconcelos MS, Sampaio AS, Hounie AG, et al. Prenatal, perinatal, and postnatal risk factors in obsessive-compulsive disorder. Biol Psychiatry. 2007;61(3):301-307.
64. Winkler-Schwartz A, Garfinkle J, Shevell MI. Autism spectrum disorder in a term birth neonatal intensive care unit population. Pediatr Neurol. 2014;51(6):776-780.
65. Yip BHK, Leonard H, Stock S, et al. Caesarean section and risk of autism across gestational age: a multi-national cohort study of 5 million births. Int J Epidemiol. 2017;46(2):429-439.
66. Zhang X, Lv CC, Tian J, et al. Prenatal and perinatal risk factors for autism in China. J Autism Dev Disord. 2010;40(11):1311-1321.

© 2019 Zhang T et al. *JAMA Network Open.*
